# Condensation of pericentrin proteins in human cells illuminates phase separation in centrosome assembly

**DOI:** 10.1242/jcs.258897

**Published:** 2021-07-26

**Authors:** Xueer Jiang, Dac Bang Tam Ho, Karan Mahe, Jennielee Mia, Guadalupe Sepulveda, Mark Antkowiak, Linhao Jiang, Soichiro Yamada, Li-En Jao

**Affiliations:** 1 Department of Cell Biology and Human Anatomy, University of California, Davis, School of Medicine, Davis, CA 95616, USA; 2 Department of Biomedical Engineering, University of California, Davis, Davis, CA 95616, USA

**Keywords:** Cell division, Centrosome maturation, Liquid-liquid phase separation

## Abstract

At the onset of mitosis, centrosomes expand the pericentriolar material (PCM) to maximize their microtubule-organizing activity. This step, termed centrosome maturation, ensures proper spindle organization and faithful chromosome segregation. However, as the centrosome expands, how PCM proteins are recruited and held together without membrane enclosure remains elusive. We found that endogenously expressed pericentrin (PCNT), a conserved PCM scaffold protein, condenses into dynamic granules during late G2/early mitosis before incorporating into mitotic centrosomes. Furthermore, the N-terminal portion of PCNT, enriched with conserved coiled-coils (CCs) and low-complexity regions (LCRs), phase separates into dynamic condensates that selectively recruit PCM proteins and nucleate microtubules in cells. We propose that CCs and LCRs, two prevalent sequence features in the centrosomal proteome, are preserved under evolutionary pressure in part to mediate liquid-liquid phase separation, a process that bestows upon the centrosome distinct properties critical for its assembly and functions.

## Abstract (precis)

**Summary:** *In situ*-tagged PCNT condenses into dynamic granules, and its coiled-coil-rich regions phase separate into centrosome-like condensates in human cells, shedding new light on centrosome assembly.

## INTRODUCTION

The centrosome acts as a major microtubule organizing center (MTOC) in many animal cells and consists of a pair of centrioles embedded in a proteinaceous network of pericentriolar material (PCM) (Conduit et al., 2015; Rieder and Borisy, 1982; Vorobjev and Chentsov Yu, 1982; Wang et al., 2011; Woodruff et al., 2014). The MTOC activity of the centrosome is determined by the PCM, which acts as a scaffold to recruit MT regulators and nucleators, such as γ-tubulin ring complexes (γ-TuRCs) (Jeng and Stearns, 1999; Moritz et al., 1995b, 1998; Oegema et al., 1999; Zheng et al., 1995). The PCM is not a static structure. In interphase cells, relatively small amounts of PCM are assembled around the centriole and organized as a layered nanometer-sized toroid (Fu and Glover, 2012; Lawo et al., 2012; Mennella et al., 2012; Sonnen et al., 2012). As the cell enters mitosis, the PCM expands dramatically into a micron-sized ensemble, with a concomitant increase in its MTOC activity as the mitotic spindle forms in a process termed centrosome maturation (Khodjakov and Rieder, 1999; Mahen and Venkitaraman, 2012; Mennella et al., 2014; Palazzo et al., 2000; Piehl et al., 2004).

Over the past decades, proteins important for centrosome maturation have been identified, and the molecular framework of centrosome maturation has been revealed (Andersen et al., 2003; Dobbelaere et al., 2008; Goshima et al., 2007; Hamill et al., 2002; Hutchins et al., 2010; Neumann et al., 2010; Sönnichsen et al., 2005; Sunkel and Glover, 1988; Woodruff et al., 2015). At the molecular level, centrosome maturation is initiated upon phosphorylation of core PCM components – e.g. spindle-defective protein 2(SPD-2)/CEP192, spindle-defective protein 5 (SPD-5), centrosomin (Cnn)/CDK5RAP2 (CEP215), and pericentrin (PCNT) – by mitotic kinases, such as Polo (Sunkel and Glover, 1988)/polo-like kinase 1 (PLK1) and aurora kinase A (Barr and Gergely, 2007; Berdnik and Knoblich, 2002; Conduit et al., 2014a; Fu and Glover, 2012; Hannak et al., 2001; Joukov et al., 2014; Kinoshita et al., 2005; Lee and Rhee, 2011; Woodruff et al., 2017, 2015; Wueseke et al., 2016). These events trigger the cooperative assembly of additional PCM proteins (Alvarez-Rodrigo et al., 2019; Chinen et al., 2021; Conduit et al., 2014b; Fu and Glover, 2012; Hamill et al., 2002; Kemp et al., 2004; Meng et al., 2015) and γ-TuRCs, leading to two mitotic centrosomes with maximized MTOC activities that facilitate bipolar spindle assembly and subsequent chromosome segregation (Chinen et al., 2021; Conduit et al., 2015; Watanabe et al., 2020; Woodruff et al., 2014). Although the mechanism of centrosome maturation has been elucidated at the molecular level, the biophysical principle of PCM assembly remains elusive at the organellar level – without an enclosing membrane, what keeps the crowded PCM proteins from dispersing?

Liquid-liquid phase separation (LLPS), a process through which macromolecules de-mix and partition from a single phase into two or more distinct phases in a concentration-dependent manner, has emerged as a mechanism that underlies a variety of cellular processes involving non-membrane-bound compartments or organelles (reviewed by Banani et al., 2017; Holehouse and Pappu, 2018; Hyman et al., 2014; Shin and Brangwynne, 2017). Recently, Woodruff et al. (2017) proposed that the centrosome is formed through LLPS. They showed that *in vitro*-purified SPD-5, a core PCM protein with extensive coiled-coils (CCs) in *Caenorhabditis elegans* (Hamill et al., 2002), forms spherical liquid ‘condensates’ *in vitro* in the presence of crowding reagents, which mimic the dense cytoplasm (Woodruff et al., 2017). SPD-5 condensates possess a centrosome-like activity *in vitro*, capable of nucleating MTs after selectively recruiting tubulin dimers and cognate proteins (ZYG-9 and TPXL-1) (Woodruff et al., 2017). These data are also consistent with the mathematical modeling of centrosomes as autocatalytic droplets formed by LLPS (Zwicker et al., 2014). However, it is unclear how closely this *in vitro* system reflects centrosomal MT nucleation *in vivo*. As Woodruff et al. (2017) did not include γ-tubulin in their study, it also remains to be determined whether SPD-5 condensates can recruit γ-tubulin, a critical *in vivo* MT nucleation factor for many species (Félix et al., 1994; Hannak et al., 2002; Joshi et al., 1992; Oakley et al., 1990; Stearns et al., 1991; Stearns and Kirschner, 1994; Zheng et al., 1991).

Contrary data suggest that LLPS may not play a role in centrosome assembly. For example, Cnn, a major mitotic PCM component and functional homolog of SPD-5 in *Drosophila melanogaster*, does not undergo dynamic internal rearrangements as it incorporates into the centrosome *in vivo* (Conduit et al., 2010, 2014a). Two short conserved domains of Cnn can self-assemble into solid-like scaffolds *in vitro*, but no liquid-to-solid phase transition has been observed (Feng et al., 2017). However, the action of these Cnn segments in the context of full-length Cnn *in vivo* remains unknown. Together, with the available evidence, it remains elusive whether LLPS underlies centrosome assembly.

In vertebrates, PCNT plays a particularly important role in PCM assembly as it is required for the initiation (Lee and Rhee, 2011; Zimmerman et al., 2004) and recruitment of key PCM components during centrosome maturation (Haren et al., 2009; Lawo et al., 2012; Zimmerman et al., 2004). We recently showed that PCNT enrichment during centrosome maturation is controlled by a co-translational targeting mechanism that ensures timely production and spatial deposition of PCNT at mitotic centrosomes (Sepulveda et al., 2018). Indeed, PCNT expression is tightly regulated. For example, human loss-of-function mutations of PCNT cause microcephalic osteodysplastic primordial dwarfism type II (Delaval and Doxsey, 2010; Griffith et al., 2008; Rauch et al., 2008), whereas elevated PCNT levels disrupt ciliary protein trafficking and sonic hedgehog signaling, and may contribute to clinical features of Down syndrome (Galati et al., 2018). Despite its importance at the cellular and organismal levels, the precise function of PCNT in centrosome assembly remains enigmatic.

Here, we demonstrate that endogenously GFP-tagged human PCNT forms droplet-like granules around centrosomes during late G2/early M phases. These GFP-PCNT granules appear to fuse and split in seconds, and are dissolved by several aliphatic alcohols, which disrupt weak hydrophobic interactions between sequences that can promote LLPS (Lin et al., 2016). These data suggest that full-length PCNT may undergo LLPS in physiologically relevant conditions during centrosome maturation. We further show that the N-terminal and middle segments of PCNT, enriched with conserved CCs and low-complexity regions (LCRs), undergo LLPS in a concentration-dependent manner with defined phase transition boundaries. Similar to dynamic pericentrosomal granules formed by the *in situ*-tagged full-length PCNT, these phase-separated PCNT ‘condensates’ are also sensitive to the same aliphatic alcohol treatment. Furthermore, condensates formed by the middle segment of PCNT transition from liquid- to gel-like states over time and exhibit centrosome-like activities in cells, including selectively recruiting endogenous PCM components and nucleating MTs. Our findings that full-length PCNT condenses into dynamic aliphatic alcohol-sensitive granules under physiologically relevant conditions, and that the CC- and LCR-rich segments of PCNT undergo concentration-dependent LLPS, shed new light on the process of LLPS and the role of CCs and LCRs, two sequence features abundant in centrosome proteome, in centrosome assembly.

## RESULTS

### Endogenously GFP-tagged PCNT forms dynamic aliphatic alcohol-sensitive pericentrosomal granules during late G2/early M phases

**Fig. 1:**
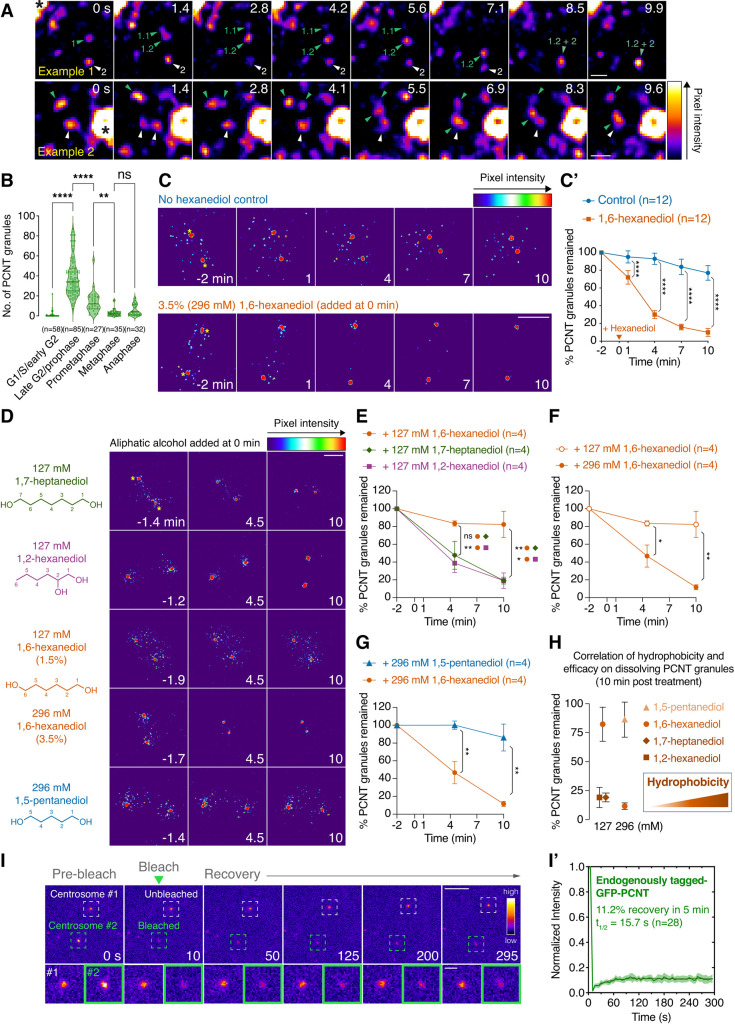
**. Endogenously GFP-tagged PCNT forms dynamic aliphatic alcohol-sensitive pericentrosomal granules during late G2/early M phases before incorporating into a largely non-dynamic mitotic PCM.** (A) Time-lapse micrographs of GFP-PCNT expressed from its endogenous locus during late G2/early M phases. Arrowheads denote the fusing and splitting events of the GFP-PCNT granules. Asterisks at time 0 denote the centrosomes. Similar results were obtained from more than three biological replicates (also see Movies 1-3). (B) Quantification of PCNT granule numbers at different cell cycle stages. Data are median±first and third quartiles. *n*, number of cells analyzed from more than three biological replicates. Representative images are shown in Fig. S2A. (C) Time-lapse micrographs of pericentrosomal GFP-PCNT granules with or without the acute 3.5%/296 mM 1,6-hexanediol treatment. Time 0 is the time of hexanediol addition. Asterisks at time −2 min denote the centrosomes. (C′) Quantification of the results shown in C. Percentage of GFP-PCNT granules remained, with or without the acute 1,6-hexanediol treatment, as the function of time was plotted and represented as mean±95% c.i. from three biological replicates. The total number of cells analyzed for each condition is indicated. The arrowhead denotes the time of hexanediol addition (time 0). (D) Time-lapse micrographs of late G2/early M cells showing pericentrosomal GFP-PCNT granules before and after acute treatments of various aliphatic alcohols at 127 or 296 mM. Note that 296 mM (or 3.5%) of 1.6-hexanediol was also the concentration used in C and C′. Time 0 is the time of aliphatic alcohol addition. Two large red dots in each frame are the centrosomes depicted, for example, by asterisks in time −1.4 min on the top panel (127 mM 1,7-heptanediol). (E-H) Quantification of the results from the experiments shown in D. After treatments of various aliphatic alcohols at 127 mM or 296 mM, the percentage of GFP-PCNT granules remained as the function of time was plotted and represented as mean±s.e.m. from two to three biological replicates. The results after 10 min of treatment are summarized in H. The relative hydrophobicity of different aliphatic alcohols is represented as an orange gradient; the darker the color, the higher the hydrophobicity approximately is. The total number of cells analyzed for each condition is indicated. (I) FRAP analysis of endogenously GFP-tagged PCNT in RPE-1 cells during late G2/early mitosis. Only one of the centrosomes (Centrosome #2) was photobleached, and the fluorescence recovery was recorded every 5 s for ∼5 min. Dashed squares delineate centrosomes. (I′) The percentage recovery and half-life (t_1/2_) after photobleaching were calculated after fitting the data with non-linear regression. Data are mean±95% c.i. *n*, number of centrosomes analyzed from two biological replicates. Statistical significance was determined by one-way ANOVA (B) or Student's *t*-test (unpaired and two-tailed) (C′,E-G). *P<0.05; **P<0.01, ****P<0.0001; ns, not significant. Scale bars: 0.5 μm (A); 5 μm (C,D); 10 μm (I); 2 μm (I, insets).

To study full-length PCNT at endogenous levels in cells, we used CRISPR technology (Lin et al., 2014a; Zhang et al., 2017) to insert super-folder GFP sequence into the 5′ end of the *PCNT* locus in hTERT-immortalized human retinal pigment epithelial (RPE-1) cells (Fig. S1). The GFP tagging at the N-terminus of PCNT did not affect PCNT expression or function during centrosome maturation, as these cells recruited PCM proteins CEP215, γ-tubulin and PCNT itself to mitotic centrosomes normally and progressed through mitosis at the same rate as the parental RPE-1 cells (Fig. S1E,F). As expected, GFP-PCNT decorated centrosomes, but upon close examination, it also formed small droplet-like granules, generally smaller than 400 nm in diameter, near centrosomes (Fig. 1A). These pericentrosomal PCNT granules were observed predominantly during late G2/early M phases, concomitant with the process of centrosome maturation (Fig. 1B; Fig. S2A). Similar pericentrosomal PCNT granules were also observed by immunostaining endogenous untagged PCNT during early mitosis (Fig. S2B). These pericentrosomal PCNT granules were highly dynamic; they appeared to fuse and split over a timescale of seconds (Fig. 1A; Movies 1, 2). Similar dynamic PCNT granules were also observed in another independent GFP knock-in clone (Movie 3). However, because the size of these granules is close to the diffraction limit of light, we could not use light microscopy to unequivocally measure their aspect ratios and determine whether they indeed have a spherical shape, a feature that would suggest a liquid form.

To probe the biophysical properties of these dynamic PCNT granules, we turned to aliphatic alcohol 1,6-hexanediol, which was originally shown to weaken the permeability barrier of nuclear pore complexes (NPCs) by disrupting weak hydrophobic interactions between phenylalanine-glycine (FG) repeats of nucleoporins (Patel et al., 2007; Schmidt and Görlich, 2015; Shulga and Goldfarb, 2003). It has also been shown that 1,6-hexanediol dissolves several membraneless liquid-like cellular assemblies, such as RNA-protein granules (e.g. P granules and stress granules) (Kroschwald et al., 2015; Updike et al., 2011). Furthermore, the liquidity of these cellular assemblies is linked to their sensitivity to 1,6-hexanediol (Kroschwald et al., 2015, 2017; Lin et al., 2016). This correlation has been attributed to the ability of 1,6-hexanediol to disrupt weak hydrophobic interactions through the hydrophobic effect exerted by its alkyl chain, as in the case for disrupting the NPC permeability barrier (Patel et al., 2007; Schmidt and Görlich, 2015; Shulga and Goldfarb, 2003), as well as the ability of 1,6-hexanediol to reduce aqueous surface tension (Romero et al., 2007).

Based on these previous studies, we treated the cells with 1,6-hexanediol acutely and immediately followed the fate of pericentrosomal PCNT granules by time-lapse microscopy. We found that PCNT granules were dissolved within minutes, whereas PCNT assembly at mitotic centrosomes was refractory to the treatment (Fig. 1C,C′). These results suggest that the stability of PCNT granules is likely maintained by weak hydrophobic interactions, and that PCNT may change its modes of interaction and/or biophysical properties upon incorporation into mitotic centrosomes, e.g. through the anchoring of PCNT near the centriolar wall via its C-terminal PACT motif (Gillingham and Munro, 2000; Takahashi et al., 2002).

We next investigated whether other aliphatic alcohols also exert similar effects on PCNT granules. We found that similar to 1,6-hexanediol, several aliphatic alcohols, varying in the length of the alkyl chain and the position of hydroxyl groups, also dissolved PCNT granules, but not the PCNT assembly at centrosomes (Fig. 1D). Furthermore, the effectiveness of the aliphatic alcohol on dissolving PCNT granules was approximately proportional to its relative hydrophobicity (Fig. 1D-H), a characteristic also observed when the NPC permeability barrier and other liquid-like membraneless cellular assemblies were exposed to various aliphatic alcohols (Lin et al., 2016; Patel et al., 2007; Ribbeck and Gorlich, 2002; Rog et al., 2017; Schmidt and Görlich, 2015; Shulga and Goldfarb, 2003; Updike et al., 2011).

We also probed the biophysical properties of the PCNT assembly at mitotic centrosomes by a fluorescence recovery after photobleaching (FRAP) experiment, in which a bleaching laser targeted the mitotic centrosome and the recovery of fluorescence was measured to quantify exchange of PCNT between the cytoplasm and the PCM. A limited fluorescence recovery of GFP-PCNT at the mitotic centrosome after photobleaching was observed (∼11% in 5 min, Fig. 1I,I′), indicating that there was little exchange of PCNT at the mitotic centrosome, and/or there was a limited amount of non-centrosomal PCNT available for exchange. Thus, consistent with the aliphatic alcohol data, this FRAP result suggests that the PCNT assembly at mitotic centrosomes is largely non-dynamic, in contrast to the nearby dynamic pericentrosomal PCNT granules. Unfortunately, due to the highly dynamic nature and the small size of pericentrosomal PCNT granules, we were unable to perform similar FRAP experiments on these PCNT granules to probe their biophysical properties.

Taken together, results from these experiments suggest that under physiologically relevant conditions and predominantly during late G2/early M phases when the centrosome is maturing, PCNT condenses into dynamic aliphatic alcohol-sensitive granules, likely through weak hydrophobic interactions between PCNT molecules. However, once PCNT is incorporated into mitotic centrosomes, the modes of interaction and/or biophysical properties of PCNT change through a yet unknown mechanism, making PCNT assembly at the PCM largely non-dynamic and resistant to the dissolution of aliphatic alcohols.

### Coiled-coils and low-complexity regions of pericentrin are more conserved than the rest of the protein sequence

**Fig. 2:**
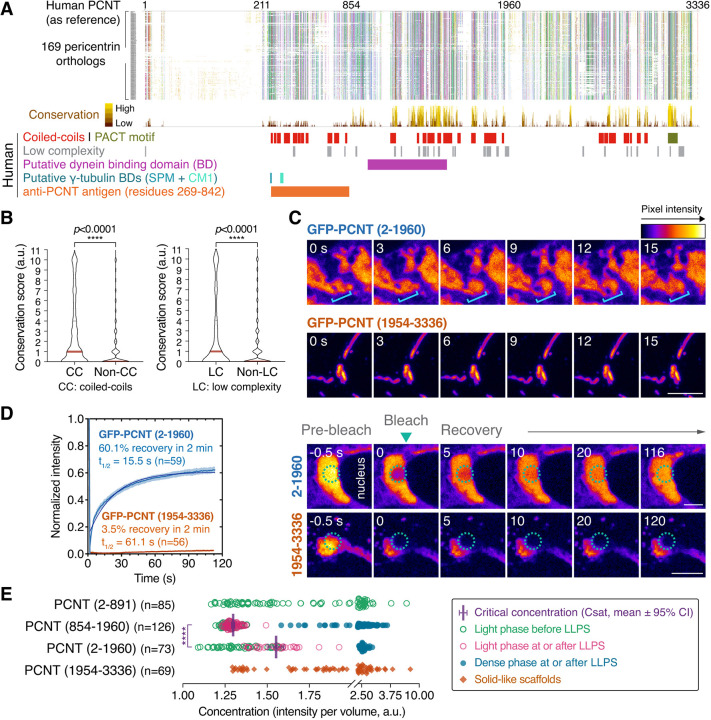
**. N-terminal segments of PCNT phase separate in a concentration-dependent manner in cells.** (A) Alignments of 169 pericentrin orthologous proteins from vertebrates (167), fruit fly (1) and budding yeast (1), colored by the ClustalX coloring scheme in Jalview (Table S1). Conservation scores, locations of the predicted CCs, PACT motif, LCRs, putative dynein and γ-tubulin binding domains (BDs) of human PCNT, and the epitopes of the anti-PCNT antibody (Abcam, ab4448) are noted below the alignments. (B) Conservation scores within or outside of CC or LCRs of human PCNT. Data are median with the third quartile. (C) Representative time-lapse micrographs of GFP-PCNT (2-1960) condensates and GFP-PCNT (1954-3336) scaffolds 24 h post Dox induction in RPE-1 cells. Brackets denote an area with dynamic rearrangement of GFP-PCNT (2-1960) condensates; PCNT (1954-3336) scaffolds are non-dynamic (also see Movies 4, 5). (D) FRAP analyses of GFP-PCNT (2-1960) condensates and GFP-PCNT (1954-3336) scaffolds in RPE-1 cells. Dashed circles denote the bleached sites. Data are mean±95% c.i. *n*, number of condensates/scaffolds analyzed from more than three biological replicates. The percentage of recovery and half-life (t_1/2_) after photobleaching were calculated after fitting the data with non-linear regression. (E) Quantification of relative protein concentrations in live cells expressing various GFP-tagged PCNT segments after Dox induction (see Fig. S4 for details and representative images). PCNT (2-1960) and PCNT (854-1960) phase separated after reaching their respective critical concentrations (Csats), the concentrations of the light phase at which LLPS just occurred. Csats are mean±95% c.i. *n*, number of cells analyzed from three biological replicates. Statistical significance was determined by the Student's *t*-test (unpaired and two-tailed). ****P<0.0001. a.u., arbitrary unit. Scale bars: 5 μm.

If condensation of PCNT is of evolutionary significance, the sequence features that contribute to condensation should be conserved across species. To test this hypothesis, we constructed an alignment of 169 pericentrin orthologous proteins – 167 from vertebrates and one each from fruit fly (D-PLP) (Martinez-Campos et al., 2004) and budding yeast (Spc110) (Knop and Schiebel, 1997; Sundberg and Davis, 1997) (Fig. 2A; Table S1). The analysis shows that a number of regions are highly conserved. One is around the C-terminus, particularly at the centrosomal anchoring PACT motif (Gillingham and Munro, 2000; Takahashi et al., 2002). Another conserved region is in the middle portion of the protein. In contrast, the N-terminus is not well conserved, with the evidence of clade-specific insertions.

To gain insights into the properties of these conserved sequences, we performed further *in silico* analyses, focusing on human PCNT (Fig. 2A). The Ncoils (Lupas et al., 1991) and SEG (Wootton, 1994) programs respectively predict that human PCNT is enriched with CCs and LCRs, which often overlap with intrinsically disordered sequences, a sequence feature that can mediate multivalent interactions to drive LLPS (Boke et al., 2016; Kato et al., 2012; Lin et al., 2015; Molliex et al., 2015; Patel et al., 2015; Riback et al., 2017) Indeed, some disorder predictors [e.g. Predictor of Natural Disordered Regions (PONDR, Peng et al., 2005, 2006)] predicted that human PCNT is largely disordered except for the C-terminal PACT motif (Fig. S3). However, as a known limitation with current disorder predictions (Atkins et al., 2015), not all disorder predictors are in complete agreement, with each predictor suggesting different degrees of disorder/order tendency [e.g. IUpred (Dosztányi et al., 2005), predicted an overall more ordered structure than PONDR]. Statistical analyses further showed that CCs and LCRs in human PCNT were significantly more conserved than non-CCs and non-LCRs (Fig. 2B). Together, these results suggest that CCs and LCRs across pericentrin orthologous proteins are likely under natural selection to preserve their molecular functions.

### The N-terminal CC/LCR-rich segments of PCNT undergo LLPS in a concentration-dependent manner

Given that both CCs and LCRs can drive LLPS (Berry et al., 2015; Boeynaems et al., 2018; Elbaum-Garfinkle et al., 2015; Fang et al., 2019; Hennig et al., 2015; Lu et al., 2020; Molliex et al., 2015; Nott et al., 2015; Rog et al., 2017; Smith et al., 2016;Wang et al., 2018; Wippich et al., 2013; Zeng et al., 2016; Zhang et al., 2018), we hypothesized that the CC/LCR-rich sequences drive LLPS of full-length PCNT to form dynamic pericentrosomal PCNT granules observed in the knock-in cells. To test this hypothesis, control PCNT transcription tightly and map LLPS determinants, we expressed GFP-tagged N- or C-terminal segments of human PCNT under the control of a doxycycline (Dox)-inducible promoter. We stably integrated each construct in RPE-1 cells using a *piggyBac* transposon system, which is free from limitations on insert size (Kim et al., 2016). Upon Dox induction, live-cell imaging showed that the N-terminal segment GFP-PCNT (2-1960) formed dynamic condensates (Fig. 2C; Movie 4) with fast internal rearrangement of molecules, as determined by FRAP (Fig. 2D). In contrast, the C-terminal segment GFP-PCNT (1954-3336) formed solid-like scaffolds with little internal rearrangement (Fig. 2C; Movie 5) or FRAP (Fig. 2D).

**Fig. 3:**
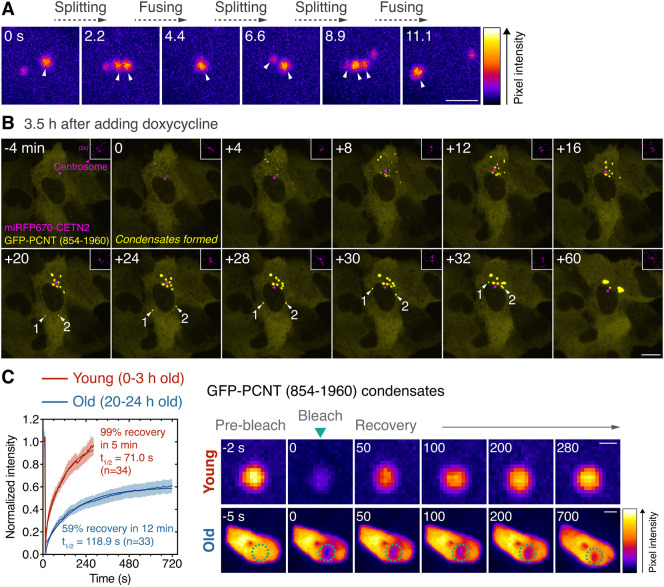
**. GFP-PCNT (854-1960) undergoes LLPS, coalesces and moves toward the centrosome.** (A) Time-lapse micrographs of GFP-PCNT (854-1960) condensates in RPE-1 cells. Arrowheads denote the fast fusing and splitting events of the spherical condensates. Similar results were obtained from three biological replicates. (B) Time-lapse micrographs of GFP-PCNT (854-1960) expressed in RPE-1 cells stably expressing miRFP670-CETN2 (insets, magenta arrowheads denote the centrosomes). Time-lapse imaging started 3.5 h post Dox induction. The time when the first condensates formed is marked as time 0. White arrowheads denote the examples of two condensates moving around the nucleus toward the centrosome. Similar results were obtained from more than three biological replicates (also see Movies 6, 7). (C) FRAP analyses of different ages of GFP-PCNT (854-1960) condensates in RPE-1 cells. Dashed circles delineate the bleached sites. Data are mean±95% c.i. *n*, number of condensates analyzed from three biological replicates. The percentage of recovery and half-life (t_1/2_) after photobleaching were calculated after fitting the data with non-linear regression. The highly mobile nature of young condensates prevented us from tracking the same condensates consistently beyond 5 min in the recovery phase of the FRAP assay. Scale bars: 2 μm (A); 10 μm (B); 1 μm (C, young condensates); 2 μm (C, old condensates).

To further map the sequences that drive LLPS, we tested GFP-tagged PCNT (2-891) and GFP-tagged PCNT (854-1960) constructs, which subdivide PCNT (2-1960) but do not disrupt individual CCs or LCRs. After inducing their expression, we compared their critical concentrations, the point above which LLPS occurs (Csat) (Asherie, 2004). To quantitatively assess the Csat in live cells, we developed an imaging and quantification strategy to measure relative protein concentrations by fluorescence intensity per volume after three-dimensional reconstruction (Fig. S4). We found that GFP-PCNT (2-891) remained diffuse in cells as its concentration increased. However, over the same concentration range, GFP-PCNT (854-1960) suddenly formed droplet-like condensates when it reached its Csat for LLPS (Fig. 2E, Fig. 3; Fig. S4). We also validated the LLPS behavior of GFP-PCNT (2-1960), which had a slightly higher Csat than GFP-PCNT (854-1960), and the lack of LLPS for GFP-PCNT (1954-3336) (Fig. 2E; Fig. S4). Importantly, as FLAG- and mScarlet-i-tagged PCNT (854-1960) also formed similar condensates (Fig. S5A,B; Movie 6), GFP tagging did not artifactually drive LLPS. Collectively, these results suggest that the abundant CCs and LCRs within PCNT (854-1960), which are well conserved across species (Fig. 2A,B), contain the key sequence elements that drive the LLPS of PCNT (2-1960) and PCNT (854-1960) segments.

### The liquidity of different PCNT assemblies is correlated with their sensitivity to 1,6-hexanediol

Thus far we had observed that two PCNT fragments (residues 2-1960 and 854-1960) undergo typical LLPS in a concentration-dependent manner to form liquid-like condensates with defined phase transitioning boundaries, at which the Csat can be determined (Fig. 2E), whereas PCNT (1954-3336) forms solid-like scaffolds in a wide range of concentrations without discernible phase transition. As the liquidity of membraneless assemblies is linked to their sensitivity to aliphatic alcohols (Kroschwald et al., 2015, 2017; Lin et al., 2016), we tested how these three assemblies would react when exposed to aliphatic alcohols by live microscopy. Upon adding 3.5% 1,6-hexanediol, the same treatment shown to dissolve pericentrosomal PCNT granules (Fig. 1C,C′), PCNT (2-1960) and PCNT (854-1960) condensates were also dissolved in minutes, whereas PCNT (1954-3336) scaffolds were not affected (Fig. S5C). Therefore, the pericentrosomal PCNT granules, formed by the *in situ*-tagged GFP-PCNT, as well as the phase-separated PCNT (2-1960) and PCNT (854-1960) condensates, are all sensitive to aliphatic alcohols in a similar manner. These results suggest that all these three cellular assemblies are liquid-like, and that the pericentrosomal PCNT granules may also be formed via LLPS, as in the case with PCNT (2-1960) and PCNT (854-1960) condensates.

### GFP-PCNT (854-1960) undergoes LLPS, coalesces and moves toward the centrosome in a dynein- and microtubule-dependent manner

**Fig. 4:**
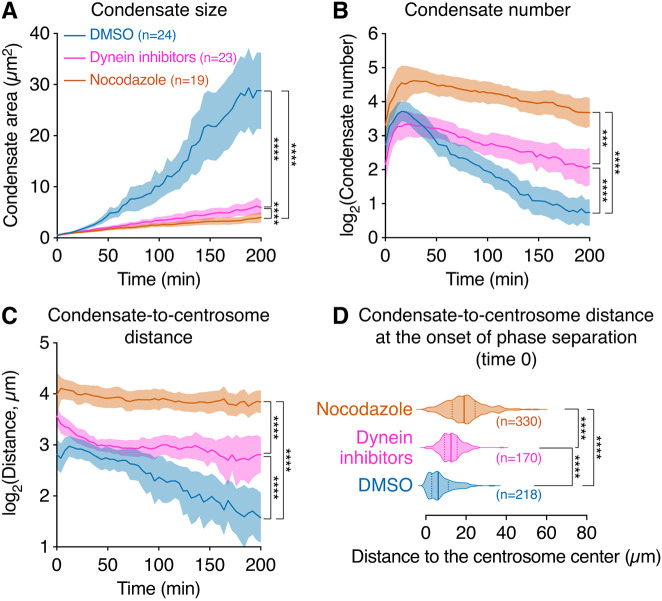
**. GFP-PCNT (854-1960) condensates coalesce and move toward the centrosome in a dynein- and MT-dependent manner.** (A-C) Quantification of the size (A), number (B), distance to the centrosome (C) and distance to the centrosome at time 0 (the start of phase separation) (D) of GFP-PCNT (854-1960) condensates over time in the cells treated with DMSO vehicle, ciliobrevin D and dynarrestin mix (50 μM each), or nocodazole (8.3 μM). GFP-PCNT (854-1960) was expressed from a Dox-inducible promoter in RPE-1 cells. Data were aligned at the onset of phase separation (time 0) of individual condensates. The fusing of PCNT (854-1960) condensates and their movement toward the centrosome were attenuated upon dynein inhibition or MT depolymerization. Initiation of phase separation also occurred closer to the centrosome with intact dynein activity and MTs (D). Data are mean±95% c.i. (A-C) or median±the first and third quartiles (D). *n*, number of cells (A-C) or condensates (D) analyzed from three (DMSO and dynein inhibition) or two (nocodazole) biological replicates. Statistical significance was determined by the *F*-test that compares the slopes of fitted lines between data sets via linear regression (A-C) or by one-way ANOVA for the distance comparison at time 0 in D. ***P=0.0001, ****P<0.0001.

Besides phase separating at a lower concentration than GFP-PCNT (2-1960) (Fig. 2E), GFP-PCNT (854-1960) condensates also exhibited different morphology and behaviors. In particular, early-stage GFP-PCNT (854-1960) condensates formed well-defined spherical liquid-like droplets as they rapidly split and fused within seconds (Fig. 3A). Over time, these GFP-PCNT (854-1960) condensates coalesced and converged at the centrosome, arcing around the nucleus in some cases, to form large pericentrosomal condensates (Fig. 3B; Movie 7). Because PCNT (854-1960) contains the putative dynein-binding domain (Tynan et al., 2000) (Fig. 2A), we tested whether the movement of PCNT (854-1960) condensates toward the centrosome is a dynein- and MT-dependent process. We treated the cells with dynein inhibitors (ciliobrevin D and dynarrestin) (Firestone et al., 2012; Höing et al., 2018) or nocodazole after Dox-induced condensate formation, and followed condensate movement by time-lapse microscopy. To quantitatively assess the effects of these treatments, we developed a Python program to semi-automatically track and calculate the size, number and distance to the centrosome (miRFP670-CETN2 labeled) of each condensate at single-cell resolution over time. When dynein was inhibited or MTs were depolymerized, fusion of the condensates, indicated by size increase and number decrease, was impaired (Fig. 4A,B), and their movements toward the centrosome were also significantly attenuated (Fig. 4C). Moreover, initial LLPS also occurred closer to the centrosome in the DMSO-treated cells than in the dynein inhibitor- or nocodazole-treated cells (Fig. 4D). Therefore, we conclude that GFP-PCNT (854-1960) and the condensates it forms move toward the centrosome in a dynein- and MT-dependent manner. As LLPS of GFP-PCNT (854-1960) takes place closer to the centrosome with intact dynein activity and MTs, this dynein- and MT-dependent transport could potentially also facilitate LLPS by concentrating and converging GFP-PCNT (854-1960) toward the centrosome along MT tracks.

### GFP-PCNT (854-1960) condensates transition from liquid- to gel-like states over time

Close examination of time-lapse data revealed that the rate of fusing and splitting decreased as PCNT (854-1960) condensates coalesced (Fig. 3B; Movies 6, 7), suggesting that the ‘liquidity’ of PCNT (854-1960) condensates decreased over time. To test his hypothesis, we used the Dox-inducible system to induce, track and analyze young (0-3 h old) and old (20-24 h old) GFP-PCNT (854-1960) condensates by FRAP. We found that young condensates recovered fluorescence almost twice as fast as the old ones (Fig. 3C). Some young condensates recovered 100% of their initial fluorescence intensity because they grew in size. These results suggest that GFP-PCNT (854-1960) condensates become ‘hardened’ over time. Such molecular aging has also been reported for other proteins that phase separate *in vitro*, such as SPD-5 (Woodruff et al., 2017), FUS (Patel et al., 2015), hnRNPA1 (Lin et al., 2015) and Tau (Wegmann et al., 2018).

### GFP-PCNT (854-1960) condensates selectively recruit endogenous PCM components

**Fig. 5:**
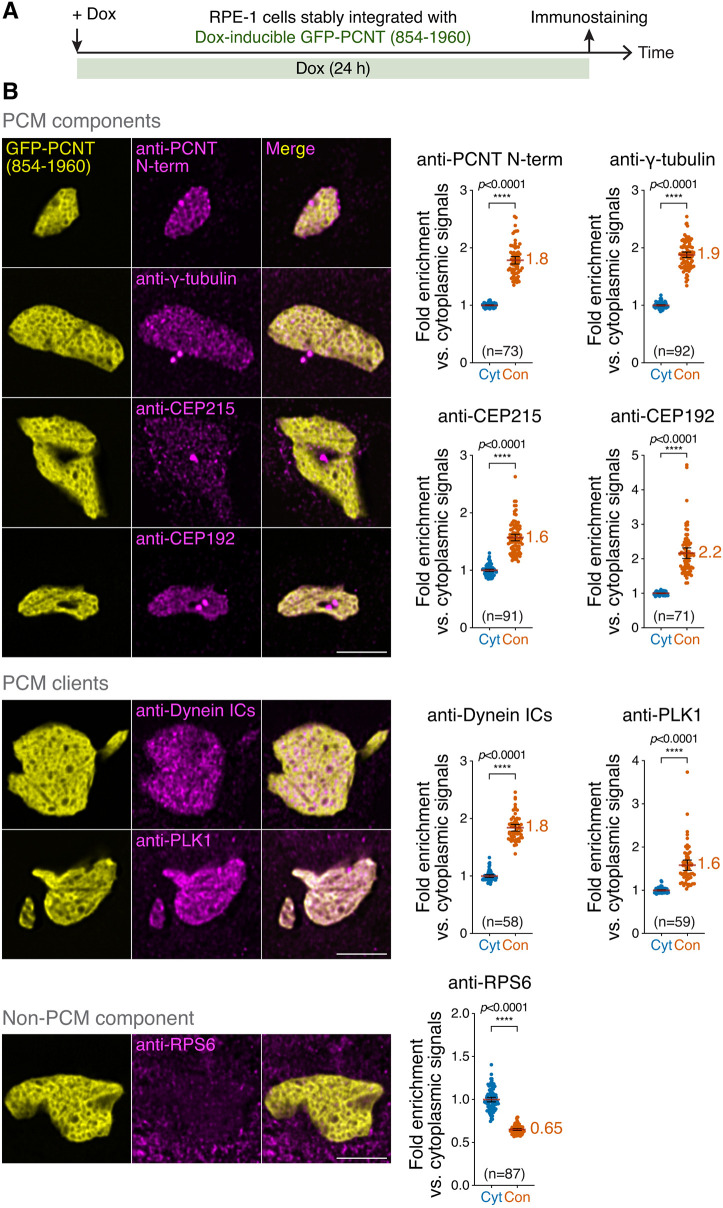
**. GFP-PCNT (854-1960) condensates selectively recruit endogenous PCM proteins and clients.** (A) Schematic of the recruitment assay to show the timeline of Dox induction and immunostaining. (B) Immunofluorescence of PCNT N-terminus (PCNT N-term), γ-tubulin, CEP215, CEP192, cytoplasmic dynein ICs (Dynein ICs), PLK1 or ribosomal protein S6 (RPS6) in RPE-1 cells after Dox induction to form GFP-PCNT (854-1960) condensates. Fold enrichment of fluorescence signals in the condensate (Con) relative to those in the cytoplasm (Cyt) was quantified. Data are mean±95% c.i. The mean of fold enrichment is noted. *n*, number of cells analyzed from at least two biological replicates per protein. Statistical significance was determined by Student's *t*-test (unpaired and two-tailed). Scale bars: 5 μm.

Because the ‘hardened’ SPD-5 condensates recruit tubulins and factors involved in MT nucleation *in vitro* (Woodruff et al., 2017), we tested whether GFP-PCNT (854-1960) condensates can also recruit PCM components, including structural (e.g. CEP215) and ‘client’ proteins (e.g. dynein and PLK1). We found that endogenous PCNT, γ-tubulin, CEP215, CEP192, dynein intermediate chains (ICs) and PLK1 were significantly enriched in GFP-PCNT (854-1960) condensates, whereas the non-PCM component ribosomal protein S6 (RPS6) was excluded (Fig. 5). Note that the antibody used to detect endogenous PCNT recognizes the epitopes before residue 854 (Fig. 2A). Thus, this antibody will not recognize PCNT (854-1960). Interestingly, these recruited proteins were not uniformly distributed in the condensate, instead forming reticular patterns that resemble mitotic PCM (Fu and Glover, 2012; Lawo et al., 2012; Mennella et al., 2012; Sonnen et al., 2012). To exclude the possibility that any phase-separated condensates could recruit PCM components, we examined the enrichment of PCM proteins in the condensates formed by HOTags, the *de novo*-designed homo-oligomeric CCs (Grigoryan et al., 2011; Huang et al., 2014; Thomson et al., 2014), which phase separate through multivalent interactions (Zhang et al., 2018). We found that PCM components γ-tubulin, CEP192 and PCNT were not enriched in the HOTag condensates (Fig. S6A-C).

Because PCNT (854-1960) condensates also recruited endogenous full-length PCNT (Fig. 5), it raised the question of whether the recruitment of other PCM components is mediated through endogenous PCNT. To test this, we repeated the recruitment assays in the presence or absence of endogenous PCNT (i.e. between the parental and *PCNT* knockout cells, Fig. S7). We found that the recruitment of PCM components to PCNT (854-1960) condensates may or may not depend on endogenous PCNT. For example, the recruitment of CEP215 was strictly dependent on endogenous PCNT (Fig. S8B, Group I), whereas the recruitment of dynein ICs and PLK1 was not (Fig. S8B, Group II). For γ-tubulin and CEP192, endogenous PCNT was not required but facilitated their recruitment to PCNT (854-1960) condensates (Fig. S8B, Group III). Taken together, these results indicate that PCNT (854-1960) condensates possess unique properties that enable them to selectively recruit endogenous PCM proteins and clients, including endogenous PCNT, which is responsible for the recruitment of some, but not all, PCM proteins to PCNT (854-1960) condensates.

### GFP-PCNT (854-1960) condensates nucleate microtubules in cells

**Fig. 6:**
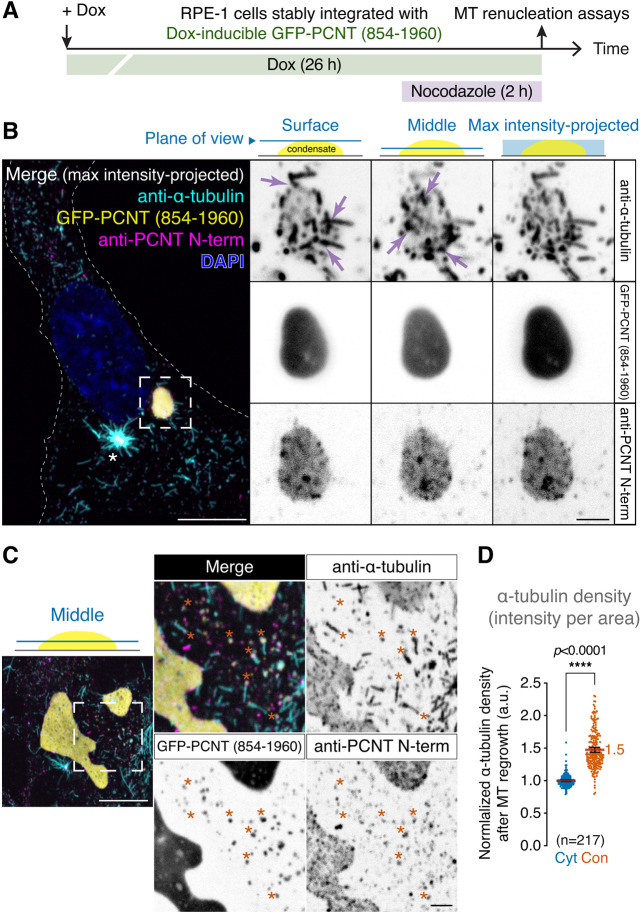
**. GFP-PCNT (854-1960) condensates nucleate MTs in cells.** (A) Schematic of the MT renucleation assay to determine whether GFP-PCNT (854-1960) condensates nucleate MTs. (B) Anti-α-tubulin immunofluorescence of the cells containing GFP-PCNT (854-1960) condensates after MT renucleation in maximum intensity-projected and single optical section views. MTs were renucleated within and on the surface of the condensate (arrows). The asterisk in the merged channels denotes the centrosome in which MT renucleation was robust. Similar MT renucleation in GFP-PCNT (854-1960) condensates was also observed in live cells (see Fig. 7; Movie 8). (C) MT renucleation also occurred in small PCNT condensates (asterisks), which also recruited endogenous PCNT. (D) Quantification of α-tubulin density (intensity per area) in GFP-PCNT (854-1960) condensates (Con) and in the surrounding cytoplasm (Cyt) after MT renucleation. Data are mean±95% c.i. The mean of fold enrichment is noted. *n*, number of condensates analyzed from three biological replicates. Statistical significance was determined by Student's *t*-test (unpaired and two-tailed). a.u., arbitrary unit. Scale bars: 10 μm and 2 μm (insets).

Because PCNT (854-1960) condensates recruit γ-tubulin (Fig. 5B; Fig. S6), the protein critical for MT nucleation (Félix et al., 1994; Hannak et al., 2002; Joshi et al., 1992; Oakley et al., 1990; Stearns et al., 1991; Stearns and Kirschner, 1994; Zheng et al., 1991), we tested whether these condensates can also nucleate MTs by MT renucleation assays (Jao et al., 2017; Sanders et al., 2017). In the MT renucleation assay, we depolymerized MTs using nocodazole, washed out the drug and monitored MT renucleation by anti-α-tubulin immunostaining (Fig. 6A). We found that MTs were renucleated not only from the centrosome as expected, but also from the interior and surface of PCNT (854-1960) condensates (Fig. 6B, arrows). The condensates also recruited endogenous PCNT as observed before (Fig. 5B; Fig. 6B). Some small PCNT (854-1960) condensates also recruited endogenous PCNT and nucleated MTs (Fig. 6C, asterisks in insets). Quantification of α-tubulin density in condensates and their surrounding cytoplasm confirmed that PCNT (854-1960) condensates had a significantly higher MT renucleation activity than the surrounding cytoplasm (Fig. 6D).

**Fig. 7:**
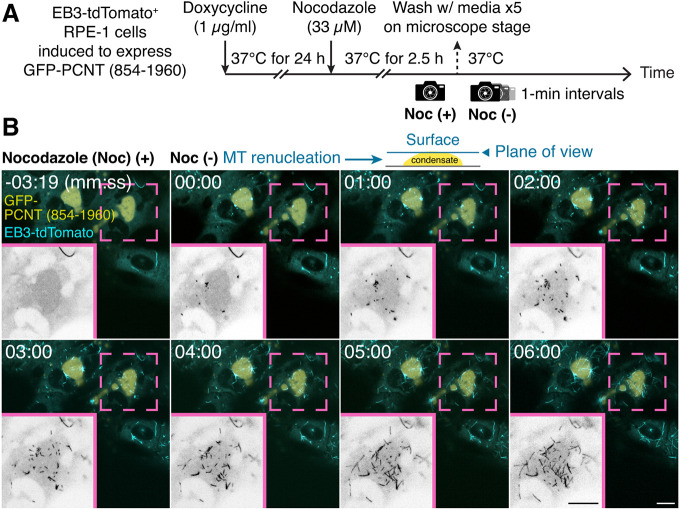
**. GFP-PCNT (854-1960) condensates nucleate MTs in live cells.** (A) Schematic of the MT renucleation assay in live cells. GFP-PCNT (854-1960) condensates in EB3-tdTomato-expressing RPE-1 cells were formed after Dox induction. MTs were then depolymerized by nocodazole (Noc). A pre-wash image was taken [Noc (+)], followed by time-lapse imaging at 1-min intervals after nocodazole was washed out on the microscope stage to follow MT renucleation [Noc (−)]. (B) Single optical sections of time-lapse micrographs of EB3-tdTomato-labeled MT plus ends on the surface of a GFP-PCNT (854-1960) condensate during MT renucleation. Time 0 was the time immediately after nocodazole was washed out (also see Movie 8). Insets show the EB3-tdTomato channel only. Similar results were obtained from three biological replicates. Scale bars: 10 μm.

We further found that the MT renucleation activity of PCNT (854-1960) condensate did not require endogenous PCNT (Fig. S8D), consistent with the results that in the absence of endogenous PCNT, many PCM components, including γ-tubulin, were still recruited to PCNT (854-1960) condensates (Fig. S8B, Groups II and III). As a negative control, we performed the MT renucleation assay in the cells with either GFP-PCNT (854-1960) or GFP-HOTag condensates. We found that only GFP-PCNT (854-1960) condensates, but not GFP-HOTag condensates, nucleated MTs (Fig. S6D-G). Thus, the GFP moiety in the fusion protein did not artifactually contribute to the MT nucleation activity of GFP-PCNT (854-1960) condensates. These results were also consistent with the observation that only PCNT condensates, but not HOTag condensates, recruited PCM components (Fig. S6A-C).

A similar MT renucleation assay was also performed in live cells, in which EB3-tdTomato was used to track the growing MT plus ends. Live-cell imaging showed that some PCNT (854-1960) condensates nucleated MTs, as EB3-tdTomato signals were emanating from the surface of PCNT condensates (Fig. 7; Movie 8). Together, from these MT renucleation assays, we conclude that GFP-PCNT (854-1960) condensates possess the centrosome-like MT nucleation activity in cells, although this activity is significantly lower than that of the centrosome (e.g. compare the MT nucleation activities between the centrosome and the condensates shown in Fig. 6B and Fig. S6E).

## DISCUSSION

Our work shows that endogenously expressed PCNT, a core PCM protein important for centrosome maturation, forms dynamic pericentrosomal granules before incorporating into mitotic centrosomes in human cells. These PCNT granules are likely formed through LLPS because (1) they are sensitive to various aliphatic alcohols that are known to disrupt phase-separated cellular assemblies (Kroschwald et al., 2017; Larson et al., 2017; Rog et al., 2017; So et al., 2019; Strom et al., 2017); (2) the CC/LCR-rich portion of PCNT undergoes concentration-dependent condensation *in cellulo* that obeys characteristics of LLPS, including a defined phase transition boundary, condensate coalescence, deformability, fast recovery in FRAP experiments and sensitivity to the 1,6-hexanediol treatment, the same treatment that also disrupts pericentrosomal PCNT granules. Recent theoretical modeling (Zwicker et al., 2014) and *in vitro* reconstitution studies (Woodruff et al., 2017) suggest that LLPS underlies centrosome assembly in *C. elegans*. To our knowledge, our study provides the first *in cellulo* evidence to support such a model and suggests that LLPS may also underlie the assembly of vertebrate centrosomes with at least one protein, PCNT, directly involved in this process.

### Is co-translational targeting of PCNT linked to its condensation during late G2/early mitosis?

The dynamic PCNT granules are predominantly observed during late G2/early mitosis, when co-translational targeting of PCNT to the centrosome peaks (Sepulveda et al., 2018). This raises an intriguing question of whether co-translational targeting facilitates the condensation of PCNT during this period. In the co-translational targeting model, multiple nascent PCNT polypeptides emerge from each polysome complex with a single large *PCNT* mRNA and are transported along the MT tracks. In principle, this process could effectively bring multiple N-terminal LLPS-driving PCNT polypeptides in close proximity. A proximity-driven LLPS can thus be envisioned, as LLPS is a concentration-dependent process. This could also explain why these dynamic PCNT granules are observed predominantly during late G2/early mitosis when PCNT production peaks. This proximity-driven phase separation model is also consistent with the results showing that the process of LLPS of the N-terminal (2-1960) and middle (854-1960) segments of PCNT can be reconstituted in a concentration-dependent manner in the cytoplasm, regardless of cell cycle stages. This model is also consistent with the observation that centrosomal targeting of PCNT (854-1960) condensates is a dynein- and MT-dependent process, and PCNT (854-1960) would phase separate closer to the centrosome with unperturbed dynein activities and intact MTs (Fig. 4D). An important future goal will be to determine whether co-translational transport of PCNT and its condensation are indeed mechanistically linked, with the former facilitating the latter.

Many phase-separated cellular assemblies contain RNA and protein (Elbaum-Garfinkle et al., 2015; Langdon et al., 2018; Lee et al., 2020; Lin et al., 2015; Maharana et al., 2018; Molliex et al., 2015; Schwartz et al., 2013; Zhang et al., 2015), and RNA can promote or inhibit phase separation (Fernandes and Buchan, 2020; Jain and Vale, 2017; Ma et al., 2021; Maharana et al., 2018). Therefore, another important future goal is to determine whether RNA – e.g. *PCNT* mRNA, ribosomal RNA in the *PCNT* polysome, or other RNA – plays an active role in the formation or regulation of pericentrosomal PCNT granules under physiological conditions.

### Functional significance of PCNT condensation

What is the physiological significance of PCNT condensation? Would it facilitate the centrosomal targeting and incorporation of PCNT? Or more broadly, is the formation of dynamic PCNT granules a prerequisite for proper centrosome assembly? Given that PCNT(854-1960) condensates move toward the centrosome in a dynein- and MT-dependent manner (Fig. 4), as in the case of co-translational targeting of *PCNT* polysomes to the centrosome (Sepulveda et al., 2018), it is tempting to speculate that by combining co-translational targeting and protein condensation with motor-mediated active transport, the cell is thus able to target PCNT (and likely other PCM proteins) to mitotic centrosomes in an efficient and ‘protected’ manner. For example, condensation could fight the force of diffusion, and this orchestrated transport along MT tracks could limit undesired interactions in the crowded cytoplasm before PCNT reaches the centrosome.

Woodruff et al. (2017) show that in the presence of crowding reagents, purified centrosomal protein SPD-5 forms liquid-like spherical condensates *in vitro*, which then rapidly ‘mature’ into a gel- or solid-like state. Strikingly, only these spherical SPD-5 condensates can nucleate MTs, but not the solid-like SPD-5 scaffolds formed in the absence of crowding reagents (Woodruff et al., 2017, 2015). Their data thus suggest that the formation of a condensate with liquid-like properties, even only transiently, might be important to allow centrosomal proteins to be properly assembled to possess the MT nucleation activity (Raff, 2019; Woodruff et al., 2017). A similar scenario could happen here – the formation of liquid-like pericentrosomal PCNT granules may enable the proper assembly of the human centrosome to organize MTs; for example, by allowing various PCM components to ‘morph’ into the desired configuration before becoming a gel/solid-like state (Fig. 1I,I′). This ‘transitioning step’ might be particularly important in forming large micron-sized membraneless assemblies, such as the mitotic PCM.

Unfortunately, the ability to directly assess the biophysical properties of these small pericentrosomal PCNT granules is limited. However, new insights into the process of PCNT/PCM assembly have been obtained from studying the PCNT (854-1960) segment and the condensate it forms *in cellulo*. This segment is one of the most conserved regions of PCNT (Fig. 2) and contains the sequence elements that drive LLPS (Fig. 3). PCNT (854-1960) condensates show a molecular aging process (Fig. 3) that resembles the possible liquid-to-gel/solid-like transition of the *in situ*-tagged GFP-PCNT granules. PCNT (854-1960) condensates also move toward the centrosome in a dynein- and MT-dependent manner (Fig. 4), similar to how the *PCNT* polysomes are co-translationally transported to the mitotic centrosome (Sepulveda et al., 2018). Morphologically, the internal organization of PCNT (854-1960) condensates, with an inhomogeneous, porous appearance (Fig. 5), also resembles that of salt-stripped mitotic centrosomes purified from flies and clams in electron tomography studies (Moritz et al., 1995a; Schnackenberg et al., 1998) in which the PCM is shown as a fibrous solid-like scaffold surrounding the centrioles. Moritz et al. (1995a) further demonstrate that upon adding bovine tubulins, MTs regrow from the PCM of the salt-stripped centrosomes, with MT nucleation sites distributed throughout the PCM and MTs oriented in different directions. Interestingly, in our MT renucleation assays, we also observed a similar MT renucleation pattern in the PCNT (854-1960) condensate; MTs were nucleated throughout the condensate and regrown into different directions (Figs 6, 7; Movie 8). PCNT (854-1960) condensates and the isolated reconstructed PCM scaffolds thus share a similar gross morphology and possess a centrosome-like MT nucleation activity. Taken together, results from our studies of the *in situ*-tagged condensing full-length PCNT and phase-separating PCNT (854-1960) segment suggest that proper centrosome function may pivot on LLPS and liquid-to-gel/solid-like phase transition during the process of centrosome assembly.

An important future goal is to rationally design phase separation-deficient (and -rescuing) PCNT variants to determine the functional significance of phase separation per se in centrosome function. It is also important to determine whether other PCM components are co-condensed with pericentrosomal PCNT granules and/or undergo a similar condensation process during centrosome assembly.

### How is PCNT (854-1960) capable of recruiting endogenous PCM components and nucleating MTs?

It is surprising that the PCNT condensate formed by only one-third of PCNT (i.e. residues 854-1960) can selectively recruit endogenous centrosomal proteins and nucleate MTs *in cellulo*. This region is particularly enriched with CCs and LCRs (Fig. 2A). However, this region does not contain the putative γ-tubulin-binding domains, which are within the first 350 residues of human PCNT (Lin et al., 2014b) (Fig. 2A), nor the CEP215-binding site, which is mapped to the C-terminus of human PCNT (residues 2390-2406) (Kim and Rhee, 2014). Yet both γ-tubulin and CEP215 (and several other PCM proteins) are recruited to the PCNT (854-1960) condensate (Fig. 5). One explanation is that their recruitment is mediated through the endogenous PCNT, which is also recruited to the condensate. Indeed, in the absence of endogenous PCNT, CEP215 is no longer recruited to the PCNT (854-1960) condensate (Fig. S8B). However, without endogenous PCNT, most of the other PCM proteins or clients we examined can still be recruited to the PCNT (854-1960) condensate – some proteins are recruited to a lesser extent (e.g. γ-tubulin and CEP192), whereas others are still recruited at a similar level as in the cells with endogenous PCNT (e.g. dynein and PLK1) (Fig. S8B). These results suggest that PCNT (854-1960) condensates recruit PCM proteins either indirectly (e.g. via the endogenous PCNT they also recruit) or directly (e.g. through yet to be identified binding sites for certain PCM proteins or clients). It is also possible that after PCNT (854-1960) phase separates, the resulting condensates gain new biophysical properties (e.g. a new binding environment) that are not present in PCNT (854-1960) monomers, thus enabling them to specifically recruit certain PCM proteins or clients. Combining mutagenesis and *in vitro* reconstitution experiments will help dissect the mechanisms underlying the selective recruitment of different PCM proteins and clients to PCNT (854-1960) condensates.

### Re-evaluating the role of coiled-coils in centrosome assembly

CCs are often enriched with low-complexity sequences (Romero et al., 1999). They are frequently predicted to be disordered as monomers but become folded upon the formation of quaternary structures (coupled folding and binding) (Anurag et al., 2012; Szappanos et al., 2010; Uversky et al., 2000). Owing to these unique properties, CCs are known to adapt vast structural variations with different superhelical stabilities to exert a wide range of biological functions (Grigoryan and Keating, 2008; Li et al., 2003; Rose and Meier, 2004).

It has long been recognized that CC proteins are enriched at the centrosome and function as parts of the ‘centromatrix’ for the recruitment of other proteins (Doxsey, 2001; Salisbury, 2003; Schnackenberg and Palazzo, 1999). Recent *in vitro* reconstitution studies of the CC PCM proteins SPD-5 (*C. elegans*) and Cnn (*D. melanogaster*) provide strong evidence to support a polymer-based mechanism of PCM assembly (Feng et al., 2017; Woodruff et al., 2017, 2015). However, the exact mechanism underlying this polymer-based assembly is still under debate. It also remains unclear whether this model is applicable to vertebrate systems (Gupta and Pelletier, 2017; Raff, 2019).

We found that in cultured human cells, not only endogenously expressed full-length PCNT condenses into dynamic granules, the CC-rich PCNT segments alone can undergo typical LLPS to form bioactive condensates with centrosome-like activities. These findings illuminate the fundamental principle of centrosome assembly and join a growing list of studies in which CC-mediated phase separation participates in a variety of biological functions (Fang et al., 2019; Lu et al., 2020; Rog et al., 2017; Vega et al., 2019; Zeng et al., 2016).

**Fig. 8:**
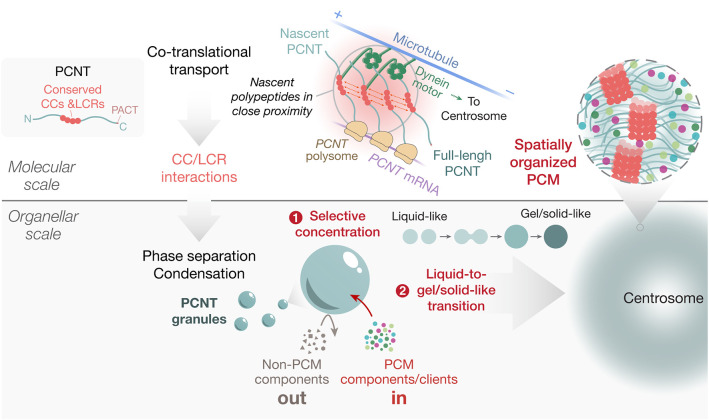
**. Model for PCNT phase separation in centrosome assembly.** PCNT is a linear multivalent protein that phase separates through its CCs and LCRs during its co-translational targeting to the centrosome when the nascent PCNT polypeptides are in close proximity in the polysome. The resulting PCNT granules/condensates promote PCM assembly by (1) selectively concentrating PCM proteins and clients; this will facilitate PCM assembly and limit the biochemical reactions at the centrosome (e.g. MT nucleation and kinase activities) from occurring elsewhere in the cytoplasm; and (2) enabling a liquid-to-gel/solid-like transitioning process during centrosome assembly; this process provides the PCM components with a thermodynamically favored pathway to assemble into a micron-sized membraneless, and yet spatially organized, PCM.

Notably, CCs and LCRs of human PCNT are enriched in the regions that are evolutionarily conserved, suggesting that these sequence features are under natural selection to preserve critical functions. We propose that PCNT is a linear multivalent protein that can undergo LLPS through its conserved CCs and LCRs to become spatially organized condensates that scaffold PCM assembly. This process is likely initiated during its co-translational targeting to the centrosome when the nascent PCNT polypeptides are in close proximity in the polysome (Fig. 8). We propose that PCNT phase separation can achieve two main goals. First, it concentrates PCM proteins and clients as the PCNT condensates selectively recruit them. This will facilitate their incorporation into the centrosome and limit the biochemical reactions at the centrosome (e.g. MT nucleation and kinase activities) from taking place elsewhere in the cytoplasm. Second, it enables a liquid-to-gel/solid-like transitioning process during centrosome assembly. This process provides the PCM proteins with a thermodynamically favorable pathway to assemble into a micron-sized membraneless, and yet spatially organized, PCM.

Although CCs could mediate other phase separation-independent activities, LLPS mediated by CC- and LCR-rich sequences observed in our study might be widespread among other CC-rich centrosomal proteins, as suggested previously (Woodruff et al., 2017). Our results encourage future studies to rethink the conceptional framework regarding CC proteins and LLPS in centrosome assembly, and to determine whether a unified mechanism is applied across metazoans.

## MATERIALS AND METHODS

### Key resources

Reagents, antibodies, cells and software used in this study are listed in Table S2.

### Constructs

To generate constructs for stable expression of GFP- or mScarlet-i-PCNT segments controlled by a Dox-inducible promoter, each PCNT segment was first amplified by PCR from pCMV-3xFLAG-EGFP-PCNT-Myc plasmid (a kind gift from Kunsoo Rhee, Seoul National University, Seoul, South Korea) (Lee and Rhee, 2011) and assembled into a vector with sfGFP or mScarlet-i by Gibson assembly (Gibson et al., 2009). The final constructs with the *piggyBac* transposon elements and Dox-inducible promoter were made by subcloning the sfGFP- or mScarlet-i-PCNT fragment into PB-TA-ERN (a gift from Knut Woltjen, Addgene, 80474) (Kim et al., 2016) using the Gateway cloning system (Thermo Fisher Scientific, Waltham, MA, USA). The following *piggyBac* transposon constructs, with amino acid sequences of human PCNT in parentheses, were used in this study: PB-TA-sfGFP-PCNT (2-891), PB-TA-sfGFP-PCNT (854-1960), PB-TA-sfGFP-PCNT (2-1960), PB-TA-sfGFP-PCNT (1954-3336) and PB-TA-mScarlet-i-PCNT (854-1960).

To make the construct for labeling MT plus ends, the EB3-tdTomato fragment was amplified by PCR from EB3-tdTomato (a gift from Erik Dent, Addgene, 50708) (Merriam et al., 2013) and cloned into a lentiviral targeting plasmid pLVX-EF1 α-mCherry-N1 without the mCherry portion (631986, Takara Bio, Mountain View, CA, USA) by Gibson assembly. The resulting plasmid, pLVX-EF1*α*-EB3-tdTomato, was used to make lentiviruses to transduce cultured cells.

To make the construct for labeling DNA, mScarlet-i-H2A construct was amplified by PCR from pmScarlet-i_H2A_C1 (a gift from Dorus Gadella, Addgene, 85053) (Bindels et al., 2017) and cloned into the same lentiviral targeting construct by Gibson assembly, as described above, to generate pLVX-EF1α-mScarlet-i-H2A. The mScarlet-i-H2A construct was also cloned into a *piggyBac* vector with the cDNA under the control of EF1*α* promoter (PB-EF1α-GW, a gift from Henry Ho, University of California, Davis, CA, USA) using the Gateway cloning system to generate PB-EF1α-mScarlet-i-H2A.

To make the construct for labeling the centrioles with far red fluorescence, the coding sequence of human centrin 2 (CETN2) was first cloned from total RNA of HEK293T cells using the SuperScript III One-Step RT-PCR System (Invitrogen). The miRFP670 was amplified by PCR from pmiRFP670-N1 (a gift from Vladislav Verkhusha, Addgene, 79987) (Shcherbakova et al., 2016). miRFP670 and CETN2 were then assembled into the same lentiviral targeting construct by Gibson assembly, as described above, to generate pLVX-EF1α-miRFP670-CETN2. The miRFP670-CETN2 construct was also cloned into PB-EF1α-GW (described above) using the Gateway cloning system to generate PB-EF1α-miRFP670-CETN2.

To generate Cas9 tagged with the nuclear localization signal (NLS) at both the N and C termini for expression in mammalian cells, an NLS from SV40 large T-antigen (Kalderon et al., 1984) was synthesized and added to hCas9, a construct encoding a human codon-optimized Cas9 (hCas9) with an NLS at its C terminus (a gift from George Church, Addgene, 41815) (Mali et al., 2013), by PCR. The final construct, pCMVSP6-NLS-hCas9-NLS-polyA_Tol2pA2, with NLS-hCas9-NLS under the control of the cytomegalovirus (CMV) immediate-early enhancer and promoter, was generated using the Gateway cloning system and the components from the Tol2kit (Kwan et al., 2007).

### Cell culture

RPE-1 cells (a gift from Irina Kaverina, Vanderbilt University, Nashville, TN, USA) were maintained in Dulbecco's modified Eagle medium/Ham's F-12 50/50 Mix (DMEM/F-12) (10-092-CV, Corning, NY, USA). HeLa cells (ATCC CCL-2, a gift from Susan Wente, Vanderbilt University, Nashville, TN, USA) and HEK293T cells (a gift from Henry Ho, University of California, Davis, CA, USA) were maintained in DMEM (10-017-CV, Corning). All cell lines were supplemented with 10% fetal bovine serum (FBS) (12303C, lot no. 13G114, Sigma-Aldrich, St. Louis, MO, USA) and 1× Penicillin Streptomycin (30–002 CI, Corning), and maintained in a humidified incubator with 5% CO_2_ at 37°C.

Cell lines used in this study were not further authenticated after obtaining from the sources. All cell lines were tested negative for mycoplasma using a PCR-based test with the Universal Mycoplasma Detection Kit (30-1012K, ATCC, Manassas, VA, USA). None of the cell lines used in this study were included in the list of commonly misidentified cell lines maintained by the International Cell Line Authentication Committee.

### Generation of GFP-PCNT knock-in cell lines

The CRISPR/Cas9 technology with a double-cut homology-directed repair (HDR) donor was used to knock-in sfGFP into the *PCNT* locus of RPE-1 cells (Lin et al., 2014a; Zhang et al., 2017). As it was unclear whether knocking in sfGFP would perturb centrosome integrity that might lead to p53-dependent cell cycle arrest (Mikule et al., 2007), making it unfavorable to isolate the knock-in clones, we first generated a *TP53* knockout RPE-1 cell line before the knock-in experiment (Fig. S7A,B). *TP53* knockout was achieved through CRISPR-mediated gene editing by co-expression of Cas9 protein with the gRNA targeting *TP53* (5′-GGGCAGCTACGGTTTCCGTC-3′) using the method described by Mali et al. (2013). The gRNA template was cloned into the gRNA Cloning Vector (Addgene, 41824) via Gibson assembly. Cas9 plasmid (1 μg) (pCMVSP6-NLS-hCas9-NLS-polyA_Tol2pA2) and gRNA plasmid (1 μg) were transfected into RPE-1 cells using Lipofectamine 3000 reagent according to the manufacturer's instructions (Invitrogen, Carlsbad, CA, USA). Cells were expanded, isolated as single colonies and screened for frameshift mutations in both *TP53* alleles by high-throughput Illumina sequencing. Sequencing data were analyzed and illustrated using the R-based toolkit CrispRVariants (Lindsay et al., 2016; Fig. S7A). The loss of TP53 expression was further confirmed by western blot analysis (Fig. S7B). A *TP53^−/−^* RPE-1 cell line (RPE-1_1-1) was used in this study (Fig. S7A,B).

To knock-in sfGFP sequence into the *PCNT* locus, crRNA/tracrRNA (i.e. the Alt-R system, Integrated DNA Technologies, Coralville, IA, USA) was used to target a sequence near the start codon of *PCNT* (CGCGCGGAGTCTGAGGGAGA). The double-cut HDR donor contains the sfGFP-PCNT cassette with 600-base pair homology arms flanked by the same guide RNA target sequence (synthesized and cloned into the pUC57-Kan vector, Genewiz, South Plainfield, NJ, USA) (Fig. S1). Annealed crRNA/tracrRNA and Cas9 protein (a kind gift from Fuguo Jiang and Jennifer Doudna; Jiang et al., 2016, 2015) were incubated in 30 mM HEPES (pH 7.5), 1 mM MgCl_2_, 200 mM KCl and 1 mM Tris (2-carboxyethyl) phosphine at 37°C for 10 min to form the Cas9 ribonucleoprotein (RNP) complex. Before electroporation, the HDR donor plasmid was mixed with 2×10^5^ of *TP53^−/−^* RPE-1 cells synchronized to early M phase using RO-3306 as performed previously (Sepulveda et al., 2018). The Cas9 RNP complexes were then mixed with the cell/donor vector mix, followed by electroporation using the Neon electroporation system with a 10-μl tip according to the manufacturer's instructions (Pulse voltage, 1200 V; pulse width, 25; pulse number ,4; Invitrogen). The final concentrations of the annealed crRNA/tracrRNA, Cas9 protein and HDR donor plasmid were 3 μM, 2 μM and 120 nM, respectively, in a total volume of 10 μl. After electroporation, the cells were grown for 10-14 days at low density. Individual clones were isolated and screened for the presence of GFP^+^ centrosomes. The GFP^+^ clones were further confirmed by anti-PCNT immunostaining and sequencing of the junctions of the sfGFP integration site (primer sequences are included in Table S3).

### Generation of PCNT knockout cell lines

Disrupting *PCNT* was achieved by electroporating the Cas9 RNP complex into *TP53^−/−^* RPE-1 cells as performed when generating the GFP-PCNT knock-in cells described above, except that no donor plasmid was included. The gRNA target sequence is near the start codon of *PCNT* (AGAGCAGCGGCGCAGAAAGG). After electroporation, the cells were grown for 10-14 days at low density. Individual clones were isolated and screened for the loss of PCNT by anti-PCNT immunostaining (Fig. S7D). The *PCNT* knockout clones were further confirmed by anti-PCNT western blot analysis (Fig. S7E).

### Generation of stable cell lines by *piggyBac* transposon-mediated integration

We used a previously described *piggyBac* transposon system (Kim et al., 2016) to generate stable cell lines that express proteins under the control of a Dox-inducible promoter (e.g. various GFP- and mScarlet-i-PCNT fusion proteins) or of the EF1α promoter (e.g. miRFP670-CETN2 and mScarlet-i-H2A fusion proteins). In brief, the *piggyBac* transposon plasmid that contains the desired transgene and a *piggyBac* transposase plasmid (PB210PA-1, System Biosciences, Palo Alto, CA, USA) were electroporated into *TP53^−/−^* RPE-1 cells simultaneously using the Neon electroporation system according to the manufacturer's instructions (Invitrogen). After 8-10 days of 200 mg/ml G418 treatment, transgene-integrated cells were screened by fluorescence signals. Sometimes, fluorescence-activated cell sorting (FACS) was further performed to obtain cells with the desired more uniform expression of the fusion proteins.

### Generation of stable cell lines by lentiviral transduction

To generate recombinant lentiviruses expressing EB3-tdTomato, pLVX-EF1α-EB3-tdTomato plasmid was co-transfected with the following third-generation packaging plasmids (gifts from Didier Trono): pMDLg/pRRE, pRSV-Rev and pMD2.G (Addgene, 12251, 12253 and 12259, respectively) (Dull et al., 1998) into HEK293T cells. Viral supernatants were collected from medium 24-48 h post transfection, filtered by a 0.22-μm filter, and were used to infect the inducible GFP-PCNT (854-1960) *TP53*^−/−^ RPE-1 cells with 8 μg/ml polybrene. Next, 18-24 h post infection, the viruses were removed, and the cells were expanded. To minimize the impact on MT dynamics, the cells expressing low levels of EB3-tdTomato (gated and collected by FACS) were used for experiments. To generate lentiviruses expressing mScarlet-i-H2A and miRFP670-CETN2 fusion proteins, the same lentiviral packaging procedure was performed as above, except for using the targeting vectors pLVX-EF1α-mScarlet-i-H2A and pLVX-EF1α-miRFP670-CETN2, respectively. The resulting viral supernatants were then used to infect the cells of interest. Sometimes, FACS was further performed to obtain cells with the desired more uniform expression of the fusion proteins.

### Immunostaining

Immunostaining was performed as described previously (Sepulveda et al., 2018; Jiang et al., 2019). In brief, cells were fixed for 15 min in 4% paraformaldehyde in 1× PBS at room temperature, or for 5 min in ice-cold 100% methanol at −20°C. Cells were then washed twice with 1× PBS and incubated with blocking solution (2% goat serum, 0.1% Triton X-100 and 10 mg/ml bovine serum albumin in 1× PBS) for 1 h at room temperature. Cells were then incubated with blocking solution containing diluted primary antibody for 1 h at room temperature, washed three times with 1× PBS and incubated with blocking solution containing diluted secondary antibody for 1 h at room temperature. Cells were washed three times with 1× PBS, and nuclei were counterstained with 0.05 mg/ml DAPI in 1× PBS for 30 min at room temperature before mounting.

### Microscopy

Microscopy was performed using a spinning disk confocal microscope system (Dragonfly, Andor Technology, Belfast, UK) with 63×/1.40 (magnification/numerical aperture) or 100×/1.40 HC PL APO objectives (Leica, Wetzlar, Germany), coupled with 1×, 1.5× or 2× motorized magnification changer. Image acquisition was controlled by Fusion software (Andor Technology) and images were captured using an iXon Ultra 888 EMCCD or Zyla sCMOS camera (Andor Technology). Sometimes, deconvolution of the images was also performed using the Fusion software (Andor Technology).

All live-cell imaging was performed with cells seeded in 35-mm glass-bottom dishes (P35G-1.5-10-C, MatTek Corp., Ashland, MA, USA or D35C4-20-1.5-N, Cellvis, Mountain View, CA, USA) mounted in a humidified chamber supplied with 5% CO_2_ inside a wrap-around environmental incubator (Okolab, Pozzuoli, Italy) with the temperature set at 37°C.

For acute treatments of live cells with aliphatic alcohols, the aliphatic alcohol was prepared in 10% FBS DMEM/F-12 medium, prewarmed to 37°C and added onto cells mounted on the microscope stage. Time-lapse microscopy was performed before and immediately after the addition of aliphatic alcohol. For the control, cells were imaged under the same acquisition conditions except that the cells were treated with 10% FBS DMEM/F-12 medium alone.

### Quantification of PCNT granule numbers at different cell cycle stages

For counting PCNT granules, confocal images of GFP-PCNT knock-in cells stably expressing mScarlet-i-H2A and miRFP670-CETN2 were converted to 8-bit color space after maximum intensity projection using Fiji (Schindelin et al., 2012). An intensity threshold was then applied to separate the GFP-PCNT granules from background signals. The number of PCNT granules was then counted using the ‘analyze particles’ function in Fiji.

The cell cycle stages of these cells were determined by analyzing the distance between centrosomes, the number of centrin dots and the DNA morphology. A G1/early S-phase cell was defined as a cell that contains one centrosome with two centrin dots and its DNA is not condensed. An S/early G2-phase cell was defined as a cell that contains two centrosomes close together (less than 1 μm apart), each with a pair of centrin dots (i.e. usually one brighter than the other, representing the mother and daughter centrioles), and its DNA is not condensed. A late G2-phase cell was defined as a cell that contains two centrosomes greater than 1 μm apart, each with a pair of centrin dots, and its DNA is not condensed, but the PCM has started to expand. A prophase cell was defined as a cell that contains condensed DNA, but the nuclear envelope is still intact. A prometaphase cell was defined as a cell that contains condensed DNA and the nuclear envelope has broken down. A metaphase cell was defined as a cell with condensed DNA aligned along the equator of the cell. An anaphase cell was defined as a cell with its condensed chromosomes just starting to segregate toward the two centrosome/spindle poles.

### Analysis of the movement of PCNT condensates upon dynein inhibition or MT depolymerization

#### Drug treatments and microscopy

To assess the movement of PCNT condensates upon dynein inhibition, the *TP53^−/−^* RPE-1 cells with stably integrated GFP-PCNT (854-1960) constructs under the control of a Dox-inducible promoter [herein named Tet-ON-GFP-PCNT (854-1960) cells] were first incubated with 1 μg/ml Dox for 2-3 h, followed by incubation with ciliobrevin D and dynarrestin mix (50 μM each), and 1 μg/ml Dox for 1 h before the start of time-lapse microscopy. To assess the movement of PCNT condensates upon MT depolymerization, a mix of 1 μg/ml Dox and 8.3 μM nocodazole was added to Tet-ON-GFP-PCNT (854-1960) cells for 2-3 h before the start of time-lapse microscopy. For the control, the same experimental procedure was performed except that the cells were treated with the DMSO vehicle alone. Cells were imaged at 4-min intervals for 6-10 h in the presence of 5% CO_2_ at 37°C for all conditions.

#### Quantification

Confocal images after maximum intensity projection were split into individual channels for GFP-PCNT (854-1960) (condensate), mScarlet-i-H2A (nucleus) and miRFP670-CETN2 (centrosome). For each channel, intensity and size thresholds were applied to identify the objects of interest, i.e. condensates, nuclei and centrosomes, as ‘masked’ objects. The ‘analyze particle’ function of Fiji was then applied to assign each masked object a unique identification (ID) number across all time frames. The (*x*,*y*) coordinates (based on the center of mass) and the area of each masked object were also calculated using Fiji.

Because ID numbers are unique, the same masked object will be represented by different ID numbers across time frames. To track the same object with different ID numbers over time, we considered that cells only moved slightly between time points with 4-min intervals, and that the nucleus was the least mobile among these three masked objects. Therefore, the ID numbers representing the same nucleus will also have the (*x*,*y*) coordinates shifted the least between any two consecutive time points. Using this feature, we developed a Python script to automatically assign a set of ID numbers to a given nucleus across timeframes so that the same nuclei could now be ‘tracked’ frame by frame. In each timeframe, this Python script also paired the condensate and centrosome objects with the nucleus of each cell in the field. Together, we were able to track all three masked objects simultaneously in each cell across timeframes. After executing the script, we manually confirmed the accuracy of the pairing process and corrected any errors. The Python script is available upon request.

Once the tracking of all three objects across timeframes was completed, the condensate size, condensate number and its distance to the centrosome in each cell over time were calculated. Data computation was performed using Pandas (Reback et al., 2020) and NumPy (Harris et al., 2020). Data visualization was performed using Matplotlib (Hunter, 2007) and GraphPad Prism (GraphPad, San Diego, CA, USA). Although phase separation occurred asynchronously, data were aligned and plotted from the start of phase separation (time 0) for any given condensate (Fig. 4).

### Fluorescence recovery after photobleaching experiments

FRAP experiments were performed using a FRAP photoablation module with a computer-controlled fiber optically pumped dye laser to bleach a region of interest (ROI) (∼2 μm in diameter) on the condensate or the centrosome after a few pre-bleach images were acquired. After photobleaching, the same ROI continued to be imaged at 2- to 5-s intervals for 5 to 12 min. Images were acquired using a Zeiss AxioObserver with a 60× objective coupled with a Yokogawa CSU-10 spinning disk confocal system anda Photometrics CoolSNAP HQ2 cooled charged-coupled device camera (BioImaging Solutions, San Diego, CA, USA). The microscope system was enclosed in an environmental chamber with the temperature set at 37°C. The photoablation and image acquisition were controlled by SlideBook software (Intelligent Imaging Innovations, Denver, CO, USA). Images and data were analyzed using ImageJ, Microsoft Excel and GraphPad Prism (GraphPad).

### Measurement of relative protein concentrations in cells

Dox-inducible cell lines expressing individual GFP-PCNT segments were seeded on glass-bottom 35-mm dishes and imaged after the addition of 1 μg/ml Dox. Imaging of different cell lines was performed with the same acquisition setting and conditions (5% CO_2_ at 37°C) using a spinning disk confocal microscope system (Dragonfly, Andor Technology). To estimate the relative protein concentrations of GFP-PCNT in cells (outlined in Fig. S4), the volume of individual condensates and their surrounding cytoplasm were first determined from confocal voxels. This was carried out by performing surface rendering of the GFP signals of the condensates (dense phase) and of the whole cytoplasm (dense and light phases) with different thresholds using the ‘surface’ reconstruction function of Imaris (Bitplane, Belfast, UK). Depending on the GFP expression level in each cell, threshold values were manually adjusted for rendering. The volume of the rendered surface and intensity sum of the GFP signals within the rendered surface were then calculated. The volume and intensity sum in the light phase were calculated by subtracting the value in the dense phase from that in the whole cytoplasm. Relative protein concentrations were calculated as intensity sum per volume. The critical concentration of phase-separated PCNT segments was determined as the concentration of the light phase when phase separation just occurred.

### Recruitment assays of proteins to the condensates

To test whether the PCNT condensate recruits PCM proteins, ∼ 5×10^4^ of Tet-ON-GFP-PCNT (854-1960) cells were seeded onto each 12-mm circular coverslip (72230-01, Electron Microscopy Sciences, Hatfield, PA, USA) in a 24-well plate and treated with 1 μg/ml Dox for 24 h to induce the formation of PCNT (854-1960) condensates. The cells were then fixed and immunostained against various PCM proteins or clients.

To compare the recruitment of PCM proteins or clients between the PCNT and non-PCNT condensates, the condensate formed by the HOTags was chosen as a non-PCNT condensate control (Zhang et al., 2018). The HOTag condensates were formed by transfecting pcDNA3-FKBP-EGFP-HOTag3 and pcDNA3-FRB-EGFP-HOTag6 plasmids (gifts from Xiaokun Shu; Addgene, 106924 and 106919, respectively) into *TP53^−/−^* RPE-1 cells using Lipofectamine 3000 reagent according to the manufacturer's instructions (Invitrogen). Next, 11-18 h post transfection, transfected cells were treated with 100 nM rapamycin for 1 h to induce the formation of HOTag condensates. In parallel, Tet-ON-GFP-PCNT (854-1960) cells were treated with 1 μg/ml Dox for 6-8 h to induce the formation of PCNT condensates, which are similar in size to the HOTag condensates at this stage, before immunostaining. In the last hour of Dox induction, 100 nM rapamycin was also added along with Dox to control for the potential effect caused by rapamycin.

To quantify the fold enrichment of various proteins in the condensate, three randomly chosen fixed areas in the condensate and in the cytoplasm per cell (2.25 μm^2^ for data in Fig. 5 or 0.159 μm^2^ for data in Fig. S6B) were selected for quantification using Fiji. The mean intensity values from the condensate or cytoplasm (from three areas each) in each cell were averaged. The fold enrichment of a given protein in the condensate is calculated as the ratio of protein intensity mean in the condensate to the overall protein intensity mean in the cytoplasm across all cells. For cells with centrosomes embedded in the PCNT condensates, PCM protein signals at the centrosomes within the PCNT condensate were excluded from quantification.

### MT renucleation assay

MT renucleation assay was adapted from previous studies (Jao et al., 2017; Sanders et al., 2017). In brief, cells were treated with 8.3 μM nocodazole for 2 h to depolymerize MTs. The 24-well plate containing the treated cells was then placed on ice and the cells were washed with ice-cold medium eight times to remove nocodazole. To allow MTs to renucleate, the plate was then placed in a 37°C water bath while the cells were incubated with prewarmed medium for 60-120 s (the optimal regrowth period varied between experiments), followed by a 10-s incubation with prewarmed extraction buffer [60 mM PIPES (pH 6.9), 25 mM HEPES, 10 mM EGTA, 2 mM MgCl_2_, 0.1% saponin, 0.25 nM nocodazole and 0.25 nM taxol]. Immediately after the extraction buffer treatment, the cells were fixed with 4% paraformaldehyde and 0.025% glutaraldehyde in cytoskeleton buffer (10 mM MES, 150 mM NaCl, 5 mM MgCl_2_, 5 mM EGTA and 5 mM D-glucose) for 10 min at room temperature. The cells were then incubated with 0.2% NaBH4 in 1× PBS for 10 min at room temperature to quench the autofluorescence before immunostaining.

For the live MT renucleation assay, the nocodazole-treated EB3-tdTomato-expressing cells with PCNT condensates were mounted on the microscope stage, first imaged in the presence of nocodazole, followed by washes with prewarmed medium five times on the microscope stage. After washes, the cells were imaged immediately again at 1-min intervals to monitor MT renucleation.

### Quantification of α-tubulin density in the MT regrowth assay

To quantify the α-tubulin density in the condensate, the condensate was first manually outlined as the first ROI using the freehand selection tool in Fiji. The second ROI was then defined as the cytoplasm space between the outline of the first ROI and the outline 1 μm larger than the first ROI. The area and intensity sum of anti-α-tubulin signals in the first (condensate) and second (cytoplasm) ROIs were then measured in Fiji. The α-tubulin density was calculated as anti-α-tubulin intensity sum per area. The normalized α-tubulin density was calculated as the ratio of α-tubulin density in the condensate to the averaged α-tubulin density in the cytoplasm. The presence of MT regrowth was also confirmed by confocal imaging.

### Multiple protein sequence alignments

Protein sequences of pericentrin orthologs from a diverse group of vertebrates, as well as two functional homologs of pericentrin from budding yeast (Spc110) and fruit fly (pericentrin-like protein, D-PLP), were retrieved from the Ensembl genome database (http://uswest.ensembl.org), resulting in a total of 233 sequences. We further filtered sequences to eliminate those with low-quality sequence reads, incomplete annotations and those inducing large gaps (e.g. due to the insertions specific to small numbers of species), resulting in a final list of 169 sequences. These 169 sequences were then aligned using MUSCLE (Edgar, 2004) and colored with the default Clustal X coloring scheme in Jalview (Waterhouse et al., 2009) (Table S1). The conservation of each residue was scored using the AMAS method of multiple sequence alignment analysis in Jalview (Livingstone and Barton, 1993).

### Statistical analysis

Statistical analysis was performed using GraphPad Prism (GraphPad). Each sample size (*n* value) is indicated in the corresponding figure or figure legend. Significance was assessed by performing Student's *t*-test, the *F*-test or one-way ANOVA, as indicated in individual figures. The experiments were not randomized. The investigators were not blinded to allocation during experiments and outcome assessment.

## Supplementary Material

Supplementary information
