# Supplementary material for: Condensation of pericentrin proteins in human cells illuminates phase separation in centrosome assembly
Source: J Cell Sci. 2021 Jul 26;134(14):jcs258897. doi: 10.1242/jcs.258897 (PMC8349556; doi:10.1242/jcs.258897)
Supplement: Supplementary information [file joces-134-258897-s1.pdf]

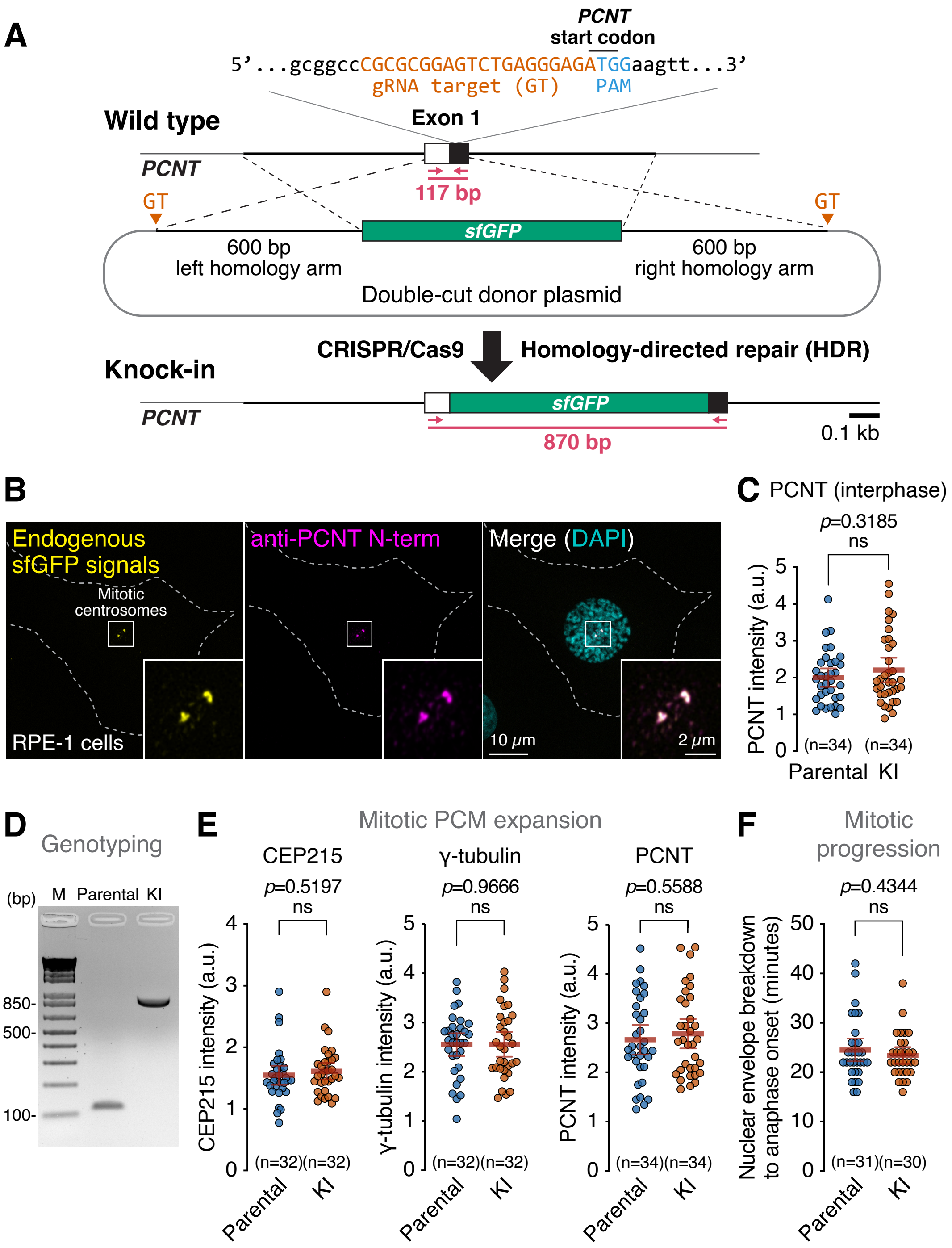

Fig. S1. Generation and validation of GFP-PCNT knock-in cells. (**A**) Schematic of CRISPR-mediated knock-in of the super-folder GFP (sfGFP) sequence into the *PCNT* locus of RPE-1 cells with a double-cut homology-directed repair donor. Arrows indicate the locations of genotyping primers. (**B**) Anti-PCNT N-terminus (N-term) immunofluorescence of a GFP-PCNT knock-in (KI) cell at prophase (determined by the DAPI-stained condensed DNA). (**C**) Quantification of PCNT levels of the parental and GFP-PCNT KI RPE-1 cells at interphase. (**D**) Genotyping results of one of the GFP-PCNT KI clones and the parental cells by PCR using the primers indicated in (A). Similar results were obtained from three biological replicates. (**E**) Quantification of CDK5RAP2/CEP215, γ-tubulin, and PCNT levels at mitotic centrosomes of the parental and GFP-PCNT KI RPE-1 cells. (**F**) Quantification of mitotic progression (the time from nuclear envelope breakdown to anaphase onset) of the parental and GFP-PCNT KI RPE-1 cells stably expressing miRFP670-CETN2 and mScarlet-i-H2A (which mark the centrosome and DNA, respectively). Data are mean ± 95% CI. Statistical significance was determined by the Student's *t*-test (unpaired and two-tailed). ns, not significant. n, number of cells analyzed from two (C and E) and three (F) biological replicates. a.u., arbitrary unit.

Journal of Cell Science • Supplementary information

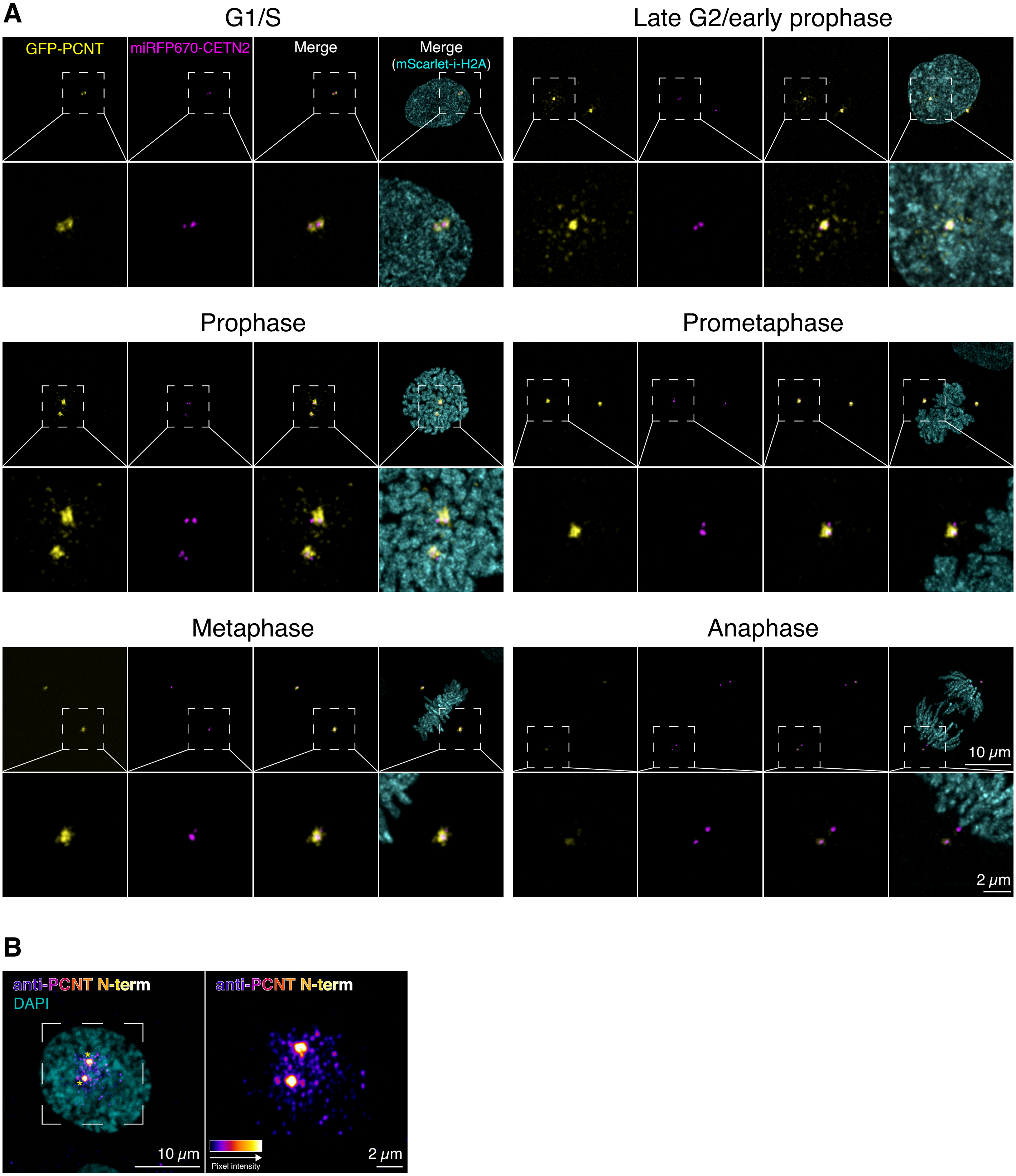

Fig. S2. In situ-tagged GFP-PCNT granules at different cell cycle stages and endogenous PCNT granules at early mitosis. (**A**) Representative confocal images of the data quantified in Fig. 1B. (**B**) Immunofluorescence of a prophase RPE-1 cell using the anti-PCNT antibody (Abcam, ab4448) to detect endogenous PCNT proteins. Note the presence of endogenous pericentrosomal PCNT granules at early mitosis. Asterisks denote the centrosomes. Similar results were obtained from three biological replicates.

Journal of Cell Science • Supplementary information

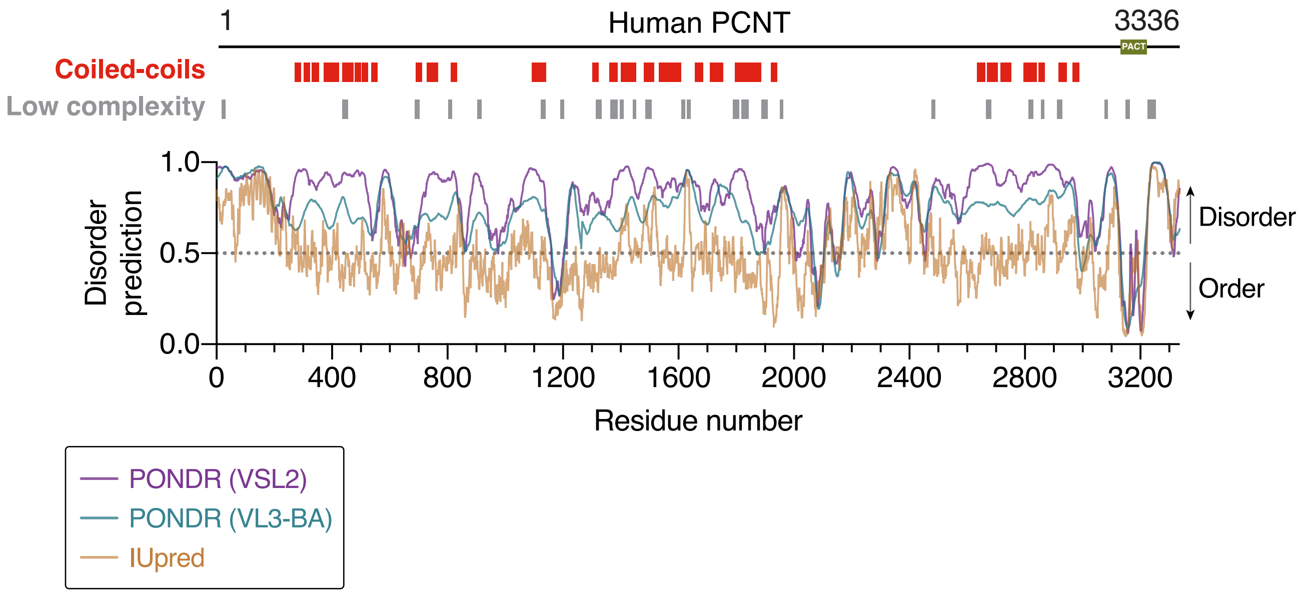

Fig. S3. Disorder predictions of human PCNT. Predicted coiled-coils and low-complexity regions of human PCNT aligned with the results of three disorder prediction algorithms (PONDR VSL2, PONDR VL3-BA, and IUpred).

Journal of Cell Science • Supplementary information

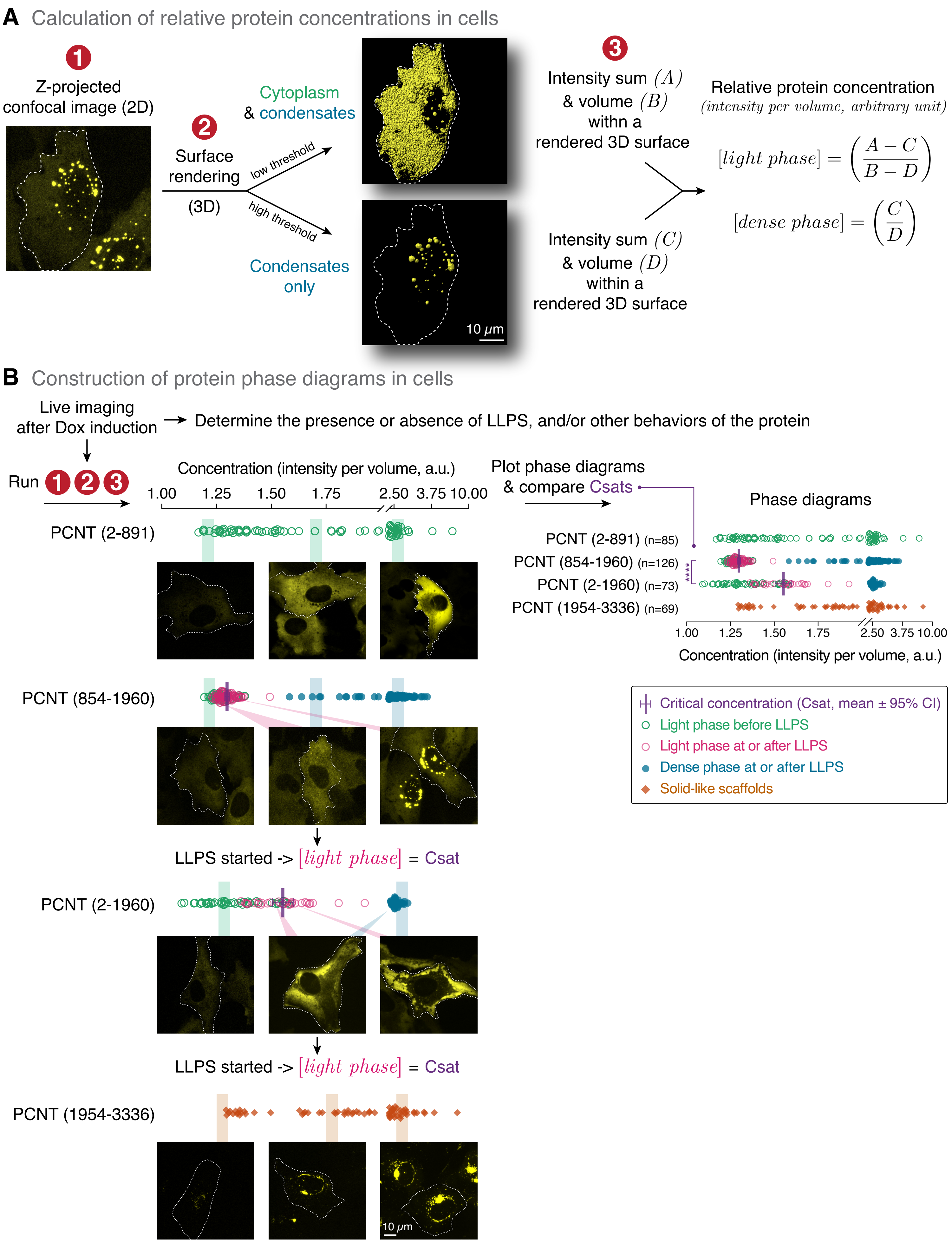
A Calculation of relative protein concentrations in cells
1
Z-projected confocal image (2D)
2
Surface rendering (3D)
low threshold
high threshold
Cytoplasm & condensates
Condensates only
10 μm
3
Intensity sum (A) & volume (B) within a rendered 3D surface
Intensity sum (C) & volume (D) within a rendered 3D surface
Relative protein concentration
(intensity per volume, arbitrary unit)
$[light\ phase] = \left(\frac{A-C}{B-D}\right)$
$[dense\ phase] = \left(\frac{C}{D}\right)$
B Construction of protein phase diagrams in cells
Live imaging after Dox induction
Determine the presence or absence of LLPS, and/or other behaviors of the protein
Run 1 2 3
Concentration (intensity per volume, a.u.)
1.00 1.25 1.50 1.75 2.50 3.75 10.00
PCNT (2-891)
PCNT (854-1960)
LLPS started -> [light phase] = Csat
PCNT (2-1960)
LLPS started -> [light phase] = Csat
PCNT (1954-3336)
10 μm
Plot phase diagrams & compare Csats
Phase diagrams
PCNT (2-891) (n=85)
PCNT (854-1960) (n=126)
PCNT (2-1960) (n=73)
PCNT (1954-3336) (n=69)
****
1.00 1.25 1.50 1.75 2.50 3.75 10.00
Concentration (intensity per volume, a.u.)
Critical concentration (Csat, mean ± 95% CI)
Light phase before LLPS
Light phase at or after LLPS
Dense phase at or after LLPS
Solid-like scaffolds

Fig. S4. Workflow of determining the relative protein concentration and critical concentration (Csat) for liquid-liquid phase separation (LLPS) in live cells (related to Fig. 2E). (A) Schematic depicts the rendering of z-stack confocal images into 3D voxels—either as the combined cytoplasmic and condensate volumes or as the condensate volumes only—using the Imaris software. The intensity sum and the volume of the rendered 3D voxels were then measured and used to calculate the relative protein concentrations in the light and dense phases. (B) Live cell imaging was used to determine the presence or absence of LLPS after Dox-induced protein expression. The relative protein concentration was calculated using the workflow described in (A). Representative confocal images were shown. The Csat is the concentration of the light phase when LLPS just started.

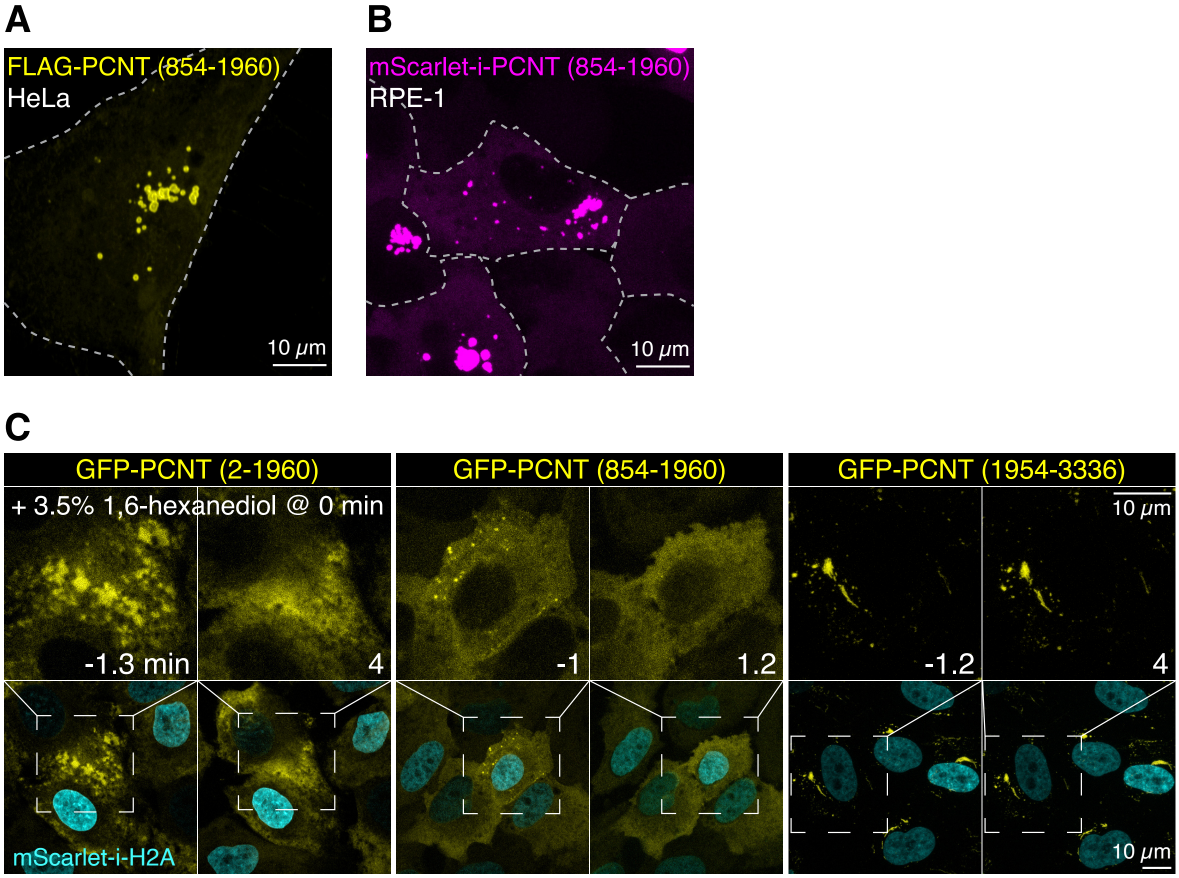

Fig. S5. Effects of epitope- and fluorescent protein-tagging and aliphatic alcohols on the behaviors of PCNT segments. (**A,B**) FLAG- and mScarlet-i-tagged PCNT (854-1960) fusion proteins form condensates. (A) Anti-PCNT N-terminus immunofluorescence of HeLa cells transiently expressing FLAG-PCNT (854-1960). Similar results were obtained from two biological replicates. (B) mScarlet-i-tagged PCNT (854-1960) formed condensates in live RPE-1 cells. Dashed lines delineate the cell boundaries. Similar results were obtained from three biological replicates. See also Movie 6. (**C**) PCNT N-terminal condensates (residues 2-1960 or 854-1960), but not the C-terminal scaffolds (residues 1954-3336) were dissolved by acute 1,6-hexanediol treatments. Representative time series of the cells containing GFP-PCNT (2-1960), GFP-PCNT (854-1960) condensates, or GFP-PCNT (1954-3336) scaffolds before and after acute treatments of 296 mM/3.5% 1,6-hexanediol. Time 0 is the time of 1,6-hexanediol addition. Similar results were observed in 4-8 cells for each condition from two to three biological replicates.

Journal of Cell Science • Supplementary information

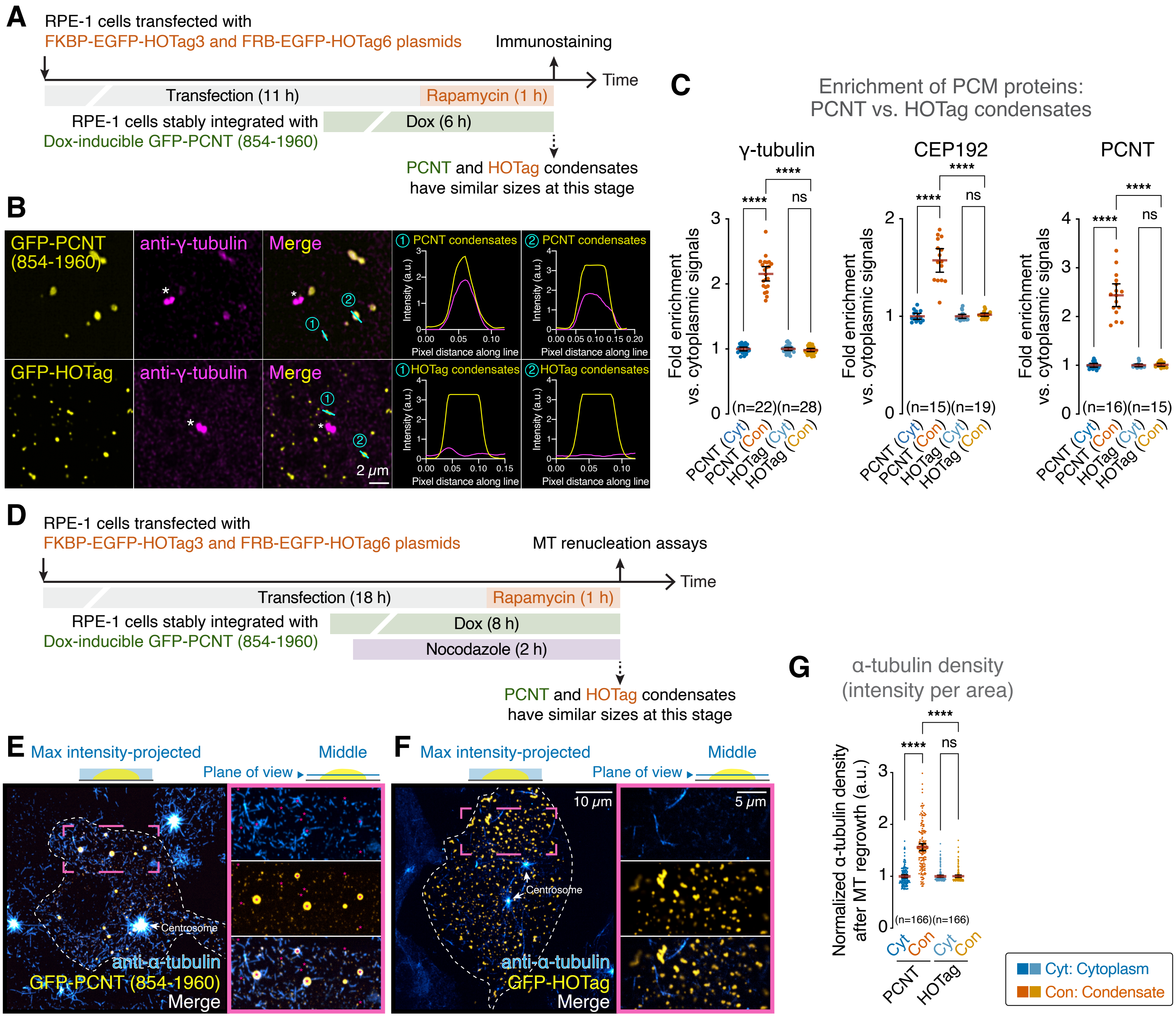

Fig. S6. HOTag condensates do not recruit PCM proteins and do not nucleate microtubules (MTs). (**A–C**) Endogenous PCM proteins are not enriched in the HOTag condensates. (A) Schematic of the recruitment assay to compare the ability of GFP-PCNT (854-1960) and GFP-HOTag condensates to recruit endogenous PCM proteins. Note that the expression of GFP-PCNT (854-1960) was only induced for 6 h (instead of 24 h in Fig. 5) so that both PCNT (854-1960) and HOTag condensates would be similar in size. (B) Representative images of anti-γ-tubulin immunofluorescence of GFP-PCNT (854-1960) and GFP-HOTag condensates. Right: The line plots of the selected regions that contain the condensates (cyan lines). Asterisks denote the centrosomes. a.u., arbitrary unit. (C) Fold enrichment of fluorescence signals in the PCNT or HOTag condensate relative to those in the cytoplasm was quantified. Data are mean ± 95% CI. n, number of cells analyzed from two, two, and one biological replicates for γ-tubulin, CEP192, and PCNT, respectively. p-values were determined by one-way ANOVA. ****: p<0.0001; ns, not significant. (**D–G**) PCNT (854-1960) condensates, but not HOTag condensates, nucleate MTs. (D) Schematic of the MT renucleation assay to compare the ability of GFP-PCNT (854-1960) and GFP-HOTag condensates to nucleate MTs. (E,F) Anti-α-tubulin immunofluorescence of the cells containing GFP-PCNT (854-1960) (E) or GFP-HOTag (F) condensates after MT renucleation in maximum intensity-projected and single optical section views. Note that MTs were renucleated from some small PCNT condensates; many short MTs also appeared to originate from the very small PCNT condensates (E, some examples depicted by asterisks). However, MT renucleation was not observed in HOTag condensates (F). In contrast, MT renucleation was robust at the centrosome as expected. (G) Quantification of α-tubulin density (intensity per area) in GFP-PCNT (854-1960) and GFP-HOTag condensates (Con) and in the surrounding cytoplasm (Cyt) after MT renucleation. Data are mean ± 95% CI. n, number of condensates analyzed from two biological replicates. Statistical significance was determined by one-way ANOVA. ****: *p*<0.0001; ns, not significant. a.u., arbitrary unit.

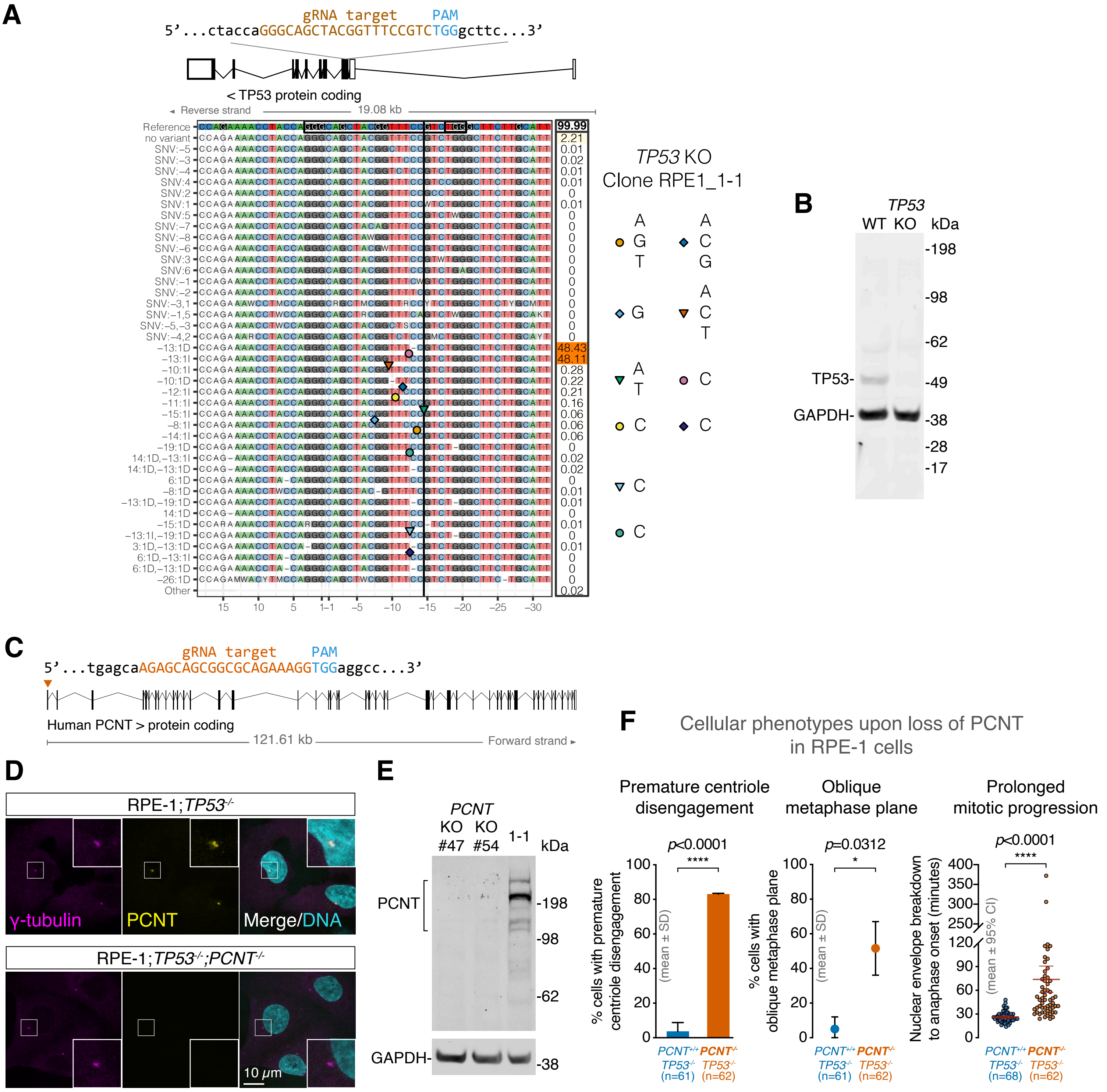

Fig. S7. Generation of *TP53* knockout and *TP53;PCNT* double knockout cells. (**A,B**) Generation of *TP53* knockout cells. (A) Schematic to show the gRNA target to disrupt *TP53* in RPE-1 cells. Illumina sequencing confirmed that one of the CRISPR-edited RPE-1 cell lines, RPE-1_1-1, has frameshift mutations at the gRNA target site in both *TP53* alleles (a 1-bp deletion and a 1-bp insertion). Sequencing data were analyzed and illustrated by an R-based toolkit, CrispRVariants (Lindsay et al., 2016). (B) Western blot analysis confirmed the loss of TP53 protein in RPE-1_1-1 cells. Anti-GAPDH staining served as the loading control. WT: parental RPE-1 cells; *TP53* KO: RPE-1_1-1 cells. (**C–F**) Generation of *TP53;PCNT* double knockout cells.(C) Schematic to show the gRNA target to disrupt *PCNT* in RPE-1 cells. (D) Loss of PCNT signals at the centrosome of a *PCNT* knockout cell line (KO#47) was confirmed by anti-PCNT immunostaining. (E) Western blot analysis confirmed the loss of PCNT protein in two *PCNT* knockout cell lines (KO#47 and KO#54). 1-1: *TP53* knockout RPE-1 cells, RPE-1_1-1. Anti-GAPDH staining served as the loading control. (F) *TP53*$^{-/-}$*;PCNT*$^{-/-}$ RPE-1 cells (e.g., KO#47 shown here) showed several cellular defects, including premature centriole disengagement, oblique metaphase plane, and prolonged mitotic progression (from nuclear envelope breakdown to anaphase onset). Data are mean ± SD or mean ± 95% CI. Statistical significance was determined by the Student's *t*-test (unpaired and two-tailed). n, number of cells analyzed from two to three biological replicates.

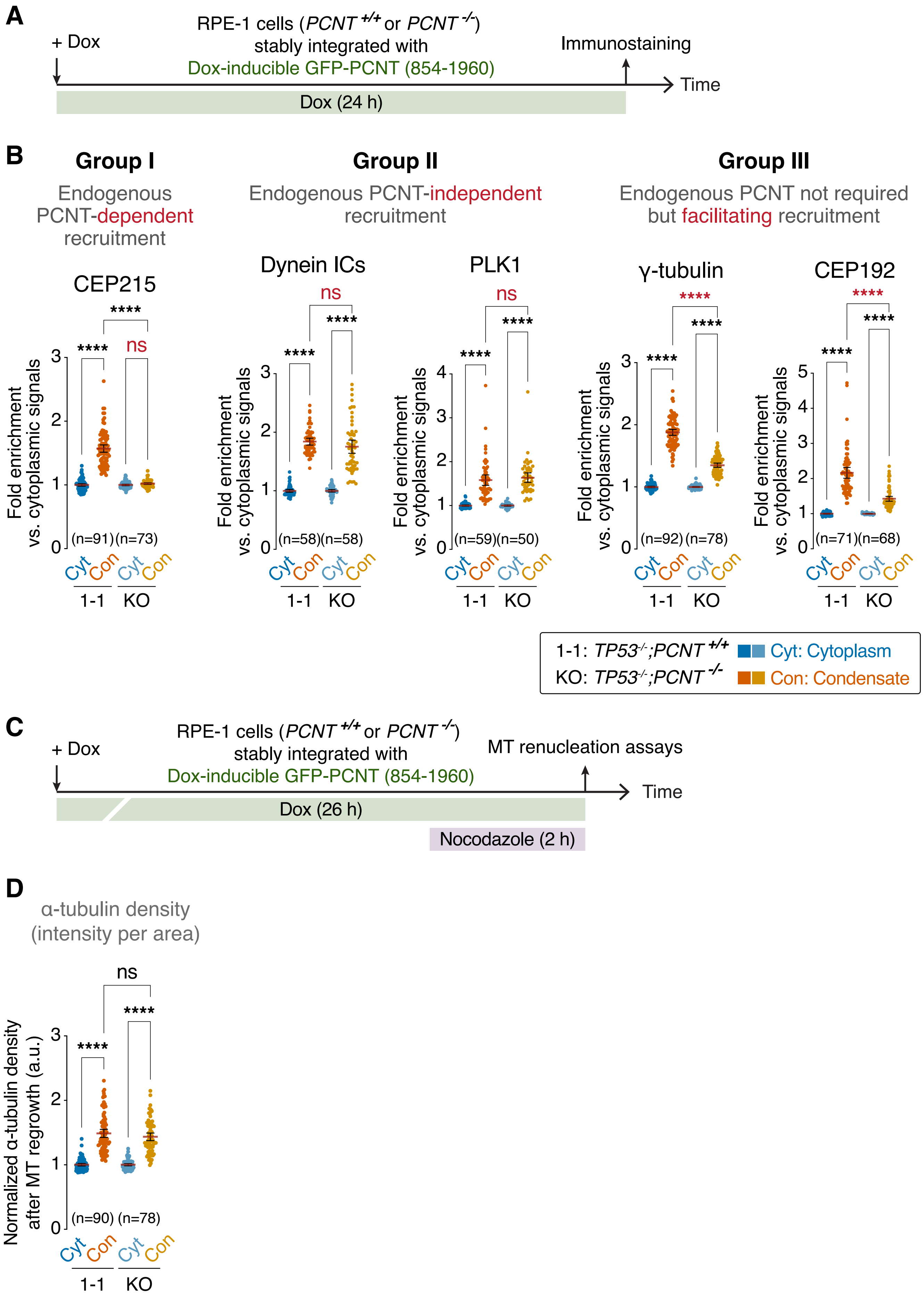

Journal of Cell Science • Supplementary information

Fig. S8. The roles of endogenous PCNT for PCNT (854-1960) condensates to recruit PCM proteins and nucleate MTs. (**A,B**) Differential requirements of endogenous PCNT for PCNT (854-1960) condensates to recruit PCM components and clients. (A) Schematic of the recruitment assay to show the timeline of Dox induction and immunostaining. (B) Fold enrichment of fluorescence signals in the PCNT (854-1960) condensate relative to those in the cytoplasm in the presence or absence of endogenous PCNT was quantified. Note that endogenous PCNT may (Group I) or may not (Group II) be required for the PCNT (854-1960) condensate to recruit endogenous PCM components and clients. In addition, endogenous PCNT may not be required for but can facilitate the recruitment of certain PCM components (Group III). (**C,D**) PCNT (854-1960) condensates can still nucleate MTs in the absence of endogenous PCNT. (C) Schematic of the MT renucleation assay to compare the ability of GFP-PCNT (854-1960) condensates to nucleate MTs in the presence or absence of endogenous PCNT. (D) Quantification of α-tubulin density (intensity per area) in GFP-PCNT (854-1960) condensates (Con) and in the surrounding cytoplasm (Cyt) during MT renucleation in the control *TP53*$^{-/-}$ (1-1) or *TP53*$^{-/-}$*;PCNT*$^{-/-}$ (KO) RPE-1 cells. Data are mean ± 95% CI. n, number of cells analyzed from two biological replicates. Statistical significance was determined by one-way ANOVA. ****: $p<0.0001$; ns, not significant. a.u., arbitrary unit.

Table S1. Coloring scheme in multiple alignments

Clustal X coloring scheme (adapted from Jalview) used in the multi-species alignments in Fig. 2A

Category	Color	Residue at position	{Threshold, Residue group}
Hydrophobic	BLUE	A, I, L, M, F, W, V	{>60%, WLVIMAFCHP}
		C	{>60%, WLVIMAFCHP}
Positive charge	RED	K, R	{>60%, KR}, {>80%, K, R, Q}
Negative charge	MAGENTA	E	{>60%, KR}, {>50%, QE},{>85%, E, Q, D}
		D	{>60%, KR}, {>85%, K, R, Q}, {>50%, ED}
Polar	GREEN	N	{>50%, N}, {>85%, N,Y}
		Q	{>60%, KR}, {>50%, QE},{>85%, Q, E, K, R}
		S, T	{>60%, WLVIMAFCHP}, {>50%, TS}, {>85%, S, T}
Cysteines	PINK	C	{>85%, C}
Glycines	ORANGE	G	{>0%, G}
Prolines	YELLOW	P	{>0%, P}
Aromatic	CYAN	H, Y	{>60%, WLVIMAFCHP}, {>85%, W, Y, A, C, P, Q, F, H, I, L, M, V}
Unconserved	WHITE	any/gap	If none of the above criteria are met

Journal of Cell Science • Supplementary information

Table S2. Key Resources Table

Reagent type	Reagent	Source	Cat. No	Additional information
Chemical compound, drug	1,7-heptanediol	Tokyo Chemical Industry Co., LTD., Tokyo, Japan	H0028	
Chemical compound, drug	1,2-hexanediol	Tokyo Chemical Industry Co., LTD., Tokyo, Japan	H0688	
Chemical compound, drug	1,6-hexanediol	MilliporeSigma, Burlington, MA	240117	
Chemical compound, drug	1,5-pentanediol	Spectrum Chemical Manufacturing Corporation, New Brunswick, NJ	P2088	
Chemical compound, drug	Ciliobrevin D	MilliporeSigma, Burlington, MA	250401	
Chemical compound, drug	Doxycycline hyclate	MilliporeSigma, Burlington, MA	D9891	
Chemical compound, drug	Dynarrestin	MilliporeSigma, Burlington, MA	SML2332	
Chemical compound, drug	Nocodazole	MilliporeSigma, Burlington, MA	M1404	
Chemical compound, drug	Rapamycin	LC Laboratories, Woburn, MA	R-5000	
Chemical compound, drug	RO-3306	R and D Systems, Minneapolis, MN	4181	
Antibody	Alexa Fluor 568 goat anti-mouse IgG [H+L]	Invitrogen, Carlsbad, CA	A11031, RRID: AB_144696	1:500 dilution
Antibody	Alexa Fluor 568 goat anti-rabbit IgG [H+L]	Invitrogen, Carlsbad, CA	A11011, RRID: AB_143157	1:500 dilution
Antibody	Alexa Fluor 647 goat anti-mouse IgG [H+L]	Invitrogen, Carlsbad, CA	A21236, RRID: AB_2535805	1:500 dilution
Antibody	Alexa Fluor 647 goat anti-rabbit IgG [H+L]	Invitrogen, Carlsbad, CA	A21245, RRID: AB_2535813	1:500 dilution
Antibody	Mouse anti-alpha-tubulin	Developmental Studies Hybridoma Bank (DSHB), University of Iowa, Iowa City, IA	12G10, RRID: AB_1157911	1:100 dilution
Antibody	Mouse anti-Dynein, 74 kDa intermediate chains, cytoplasmic, clone 74.1	EMD Millipore, Burlington, MA	MAB1618, RRID: AB_2246059	1:500 dilution
Antibody	Mouse anti-hGAPDH	Developmental Studies Hybridoma Bank (DSHB), University of Iowa, Iowa City, IA	DSHB-hGAPDH-2G7, RRID: AB_2617426	1:300 dilution
Antibody	Mouse anti-PLK1	EMD Millipore, Burlington, MA	05-844, RRID: AB_310836	1:1000 dilution
Antibody	Mouse anti-p53	Abcam, Cambridge, MA	ab1101, RRID: AB_297667	1:1000 dilution
Antibody	Mouse anti-ribosomal protein S6 (C-8)	Santa Cruz Biotechnology Inc., Santa Cruz, CA	sc-74459, RRID: AB_1129205	1:500 dilution
Antibody	Mouse anti-γ-tubulin	MilliporeSigma, Burlington, MA	T6557, RRID: AB_477584	1:500 dilution
Antibody	Rabbit anti-CDK5RAP2	EMD Millipore, Burlington, MA	06-1398, RRID: AB_11203651	1:500 dilution
Antibody	Rabbit anti-CEP192	Bethyl, Montgomery, TX	A302-324A, RRID: AB_1850234	1:500 dilution
Antibody	Rabbit anti-PCNT	Abcam, Cambridge, MA	ab4448, RRID: AB_304461	1:1000 dilution
Commercial system or kit	Lipofectamine™ 3000 Transfection Reagent	Invitrogen, Carlsbad, CA	L3000-008	
Commercial system or kit	Neon™ Transfection System	Invitrogen, Carlsbad, CA	MPK5000	
Cell line	HEK293T cells	A gift from Henry Ho, University of California, Davis, Davis, CA	CRL-3216, RRID: CVCL_0063	
Cell line	HeLa cells	A gift from Susan Wente, Vanderbilt University, Nashville, TN	CCL-2, RRID: CVCL_0030	
Cell line	hTERT RPE-1 cells	A gift from Irina Kaverina, Vanderbilt University, Nashville, TN	CRL-4000, RRID: CVCL_4388	
		Johannes Schindelin, Albert Cardona, Pavel Tomancak		

Software	Fiji (ImageJ)		RRID: SCR_002285
Software	Fusion	Andor Technology, Belfast, UK	
Software	Imaris	Bitplane, Belfast, UK	RRID: SCR_007370
Software	Jalview	The Barton Group, University of Dundee, Scotland, UK	RRID: SCR_006459
Software	GraphPad Prism	Graphpad, San Diego, CA	RRID: SCR_002798
Software	Python	Python Software Foundation	RRID: SCR_008394
Software	R	R Project for Statistical Computing	RRID: SCR_001905

Table S3. Oligo sequences

Genotyping primers for screening for GFP-PCNT knock-in clones shown in Fig. S1A

Sequence (5' to 3')
gagcgaaggctgctctgtgt
cttcgtcctcccggcctcca

Journal of Cell Science • Supplementary information

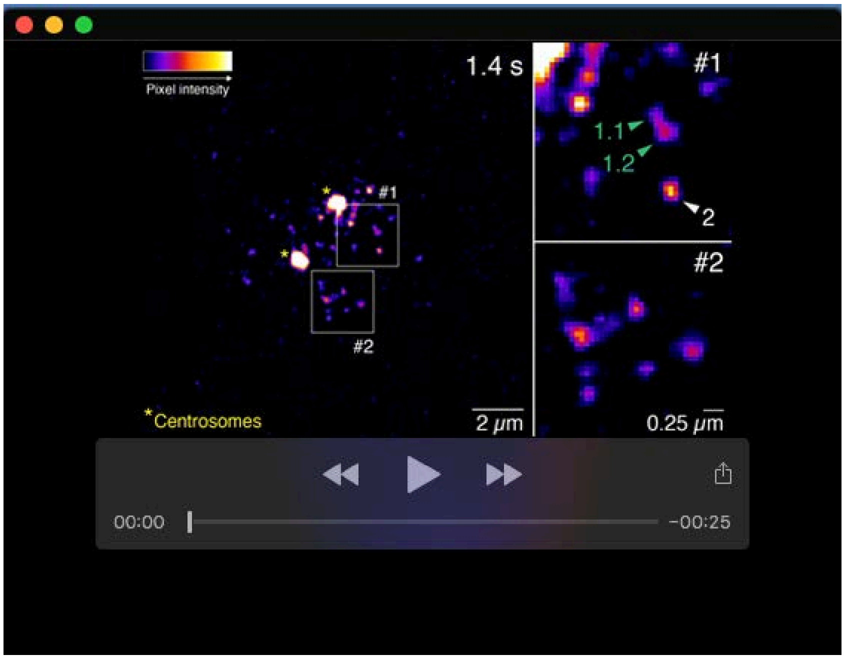

Movie 1. Time-lapse movie of a GFP-PCNT knock-in cell. Selected time-lapse micrographs (inset #1) are shown in Example 1 of Fig. 1A. Arrowheads denote the fusing and splitting events of the GFP-PCNT granules. Asterisks denote the centrosomes.

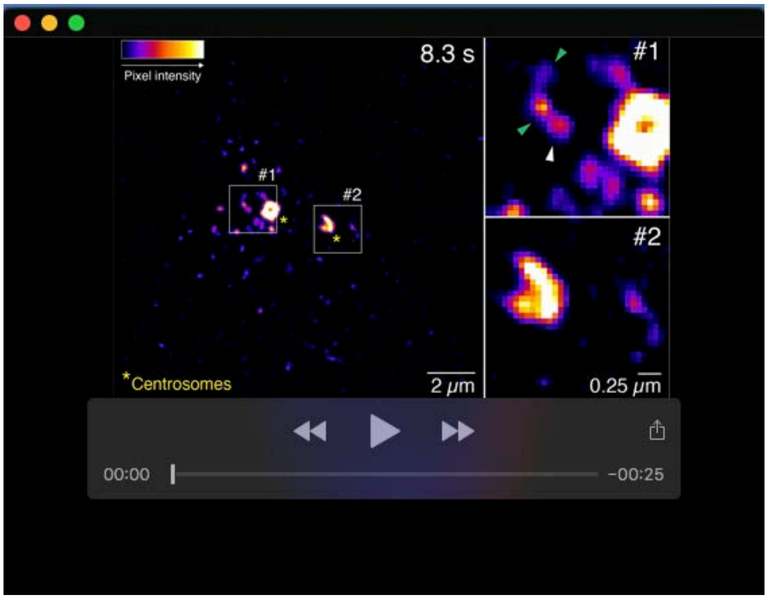

Movie 2. Time-lapse movie of a GFP-PCNT knock-in cell. Selected time-lapse micrographs (inset #1) are shown in Example 2 of Fig. 1A. Arrowheads denote the fusing and splitting events of the GFP-PCNT granules. Asterisks denote the centrosomes.

Journal of Cell Science • Supplementary information

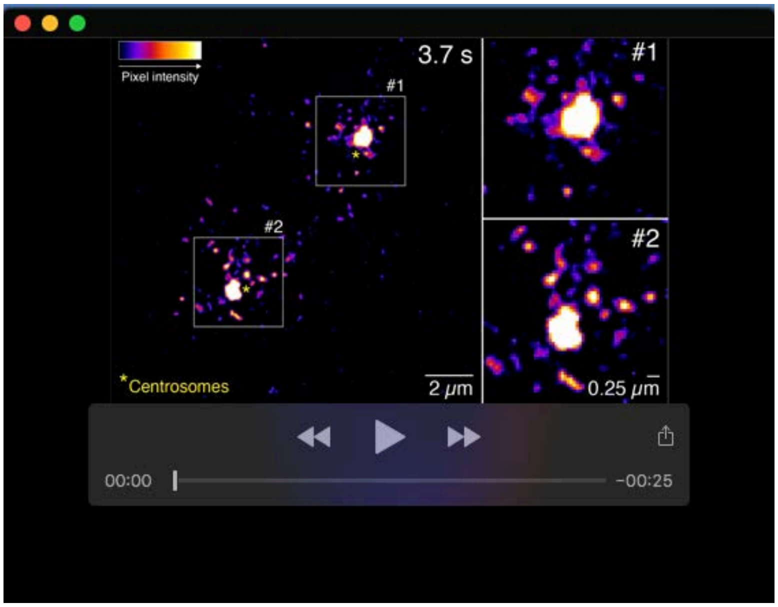

Movie 3. Time-lapse movie of a GFP-PCNT knock-in cell from another independent knock-in clone. Asterisks denote the centrosomes. Note the fusing and splitting events of the GFP-PCNT granules near centrosomes (asterisks).

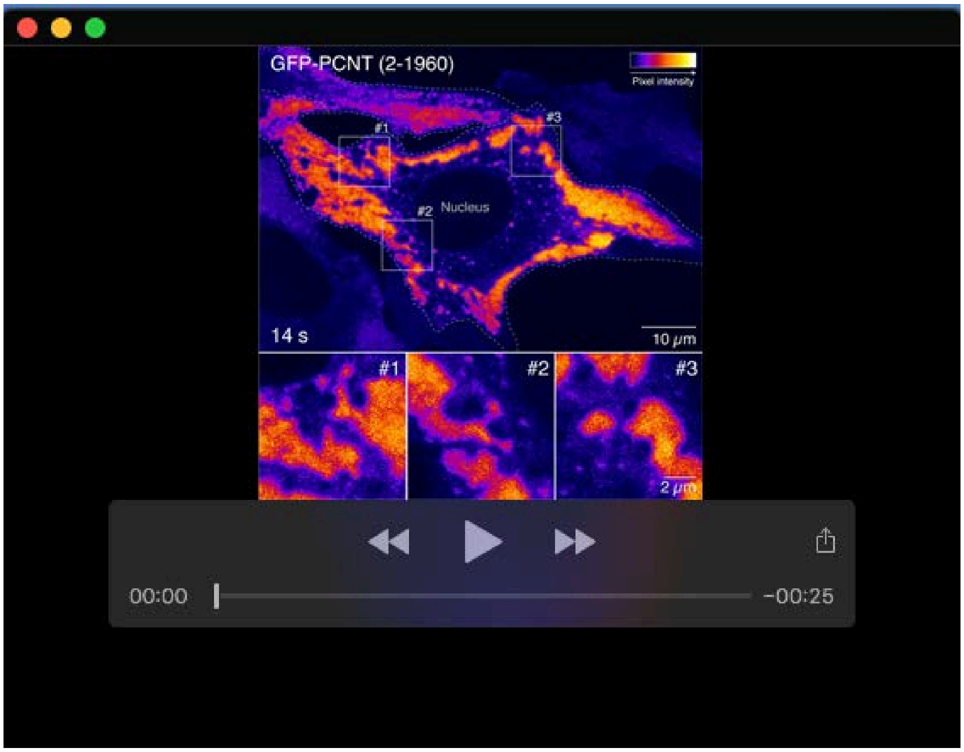

Movie 4. Time-lapse movie of GFP-PCNT (2-1960) condensates in RPE-1 cells 24 h post Dox induction. Selected time-lapse micrographs (inset #1) are shown in Fig. 2C.

Journal of Cell Science • Supplementary information

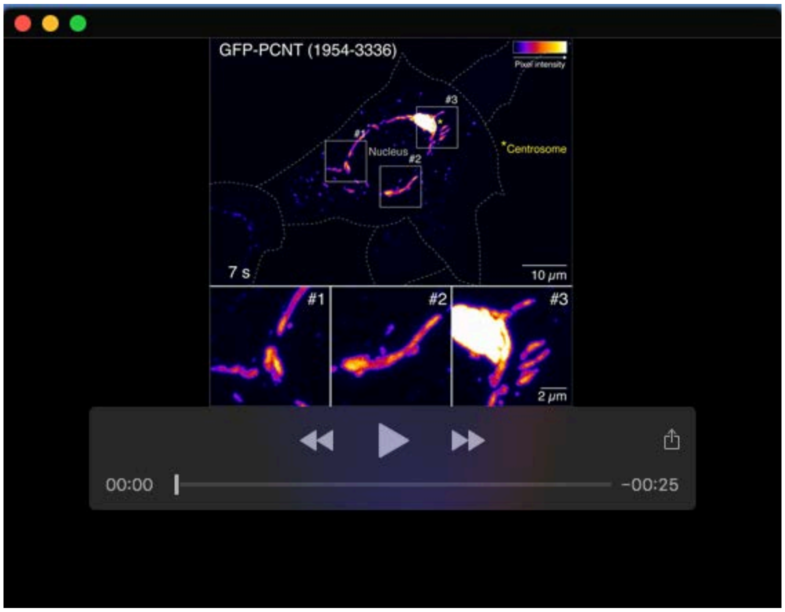

Movie 5. Time-lapse movie of GFP-PCNT (1954-3336) solid-like scaffolds in RPE-1 cells 24 h post Dox induction. Selected time-lapse micrographs (inset #1) are shown in Fig. 2C.

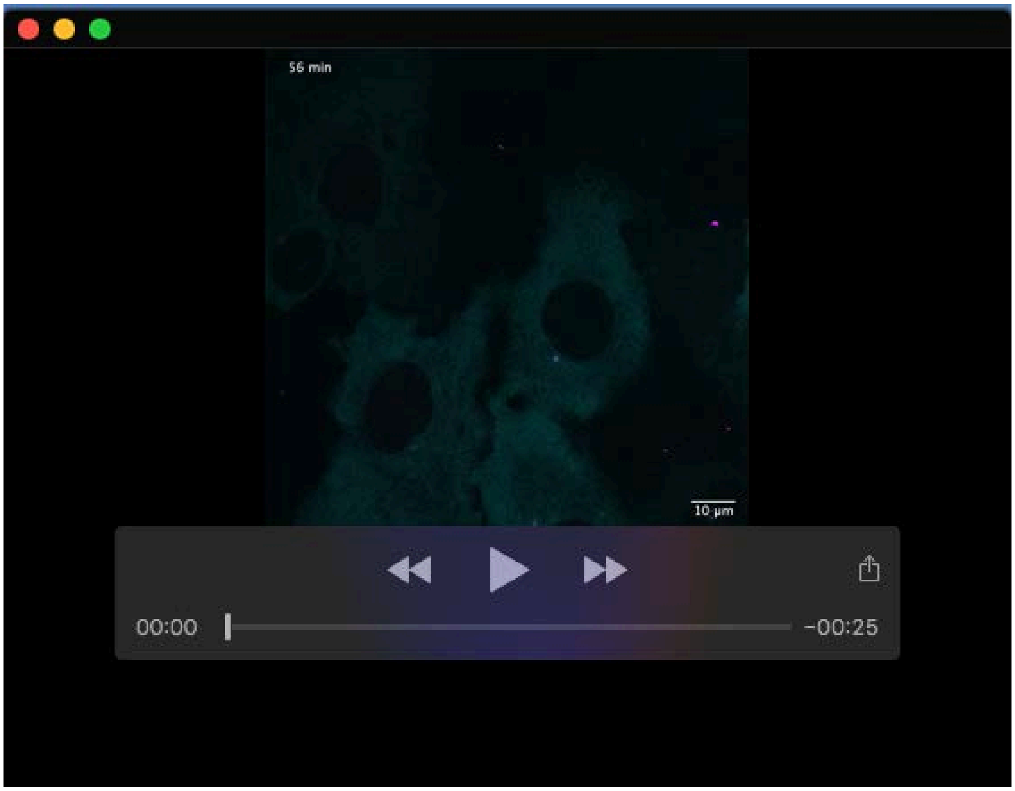

Movie 6. Time-lapse movie of mScarlet-i-PCNT (854-1960) undergoing liquid-liquid phase separation in RPE-1 cells. Note the concentration-dependent formation of mScarlet-i-PCNT (854-1960) condensates (cyan), the convergence of condensates, and the movement of condensates toward the centrosome (labeled by miRFP670-CETN2, magenta).

Journal of Cell Science • Supplementary information

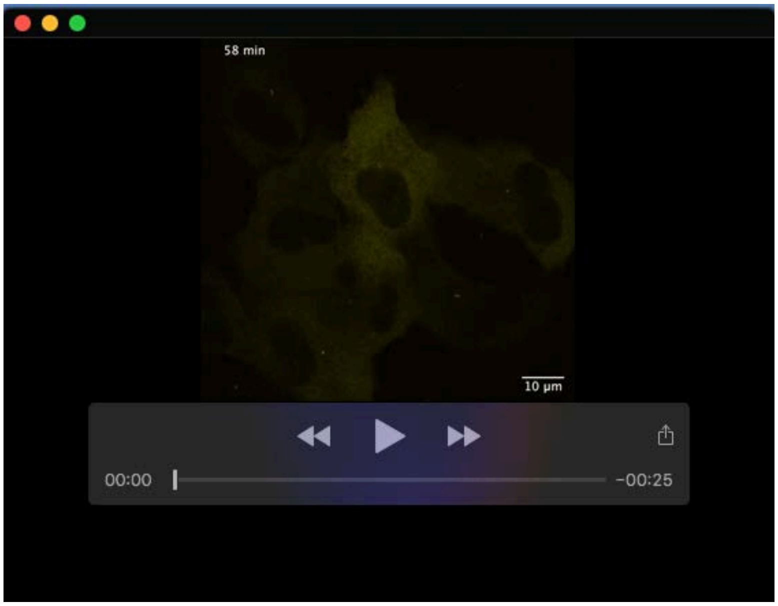

Movie 7. Time-lapse movie of GFP-PCNT (854-1960) undergoing liquid-liquid phase separation in RPE-1 cells. Time-lapse imaging started 3.5 h post Dox induction. Note the concentration-dependent formation of GFP-PCNT (854-1960) condensates (yellow), the convergence of condensates, and the movement of condensates toward the centrosome (labeled by miRFP670-CETN2, magenta). Selected time-lapse micrographs are shown in Fig. 3B.

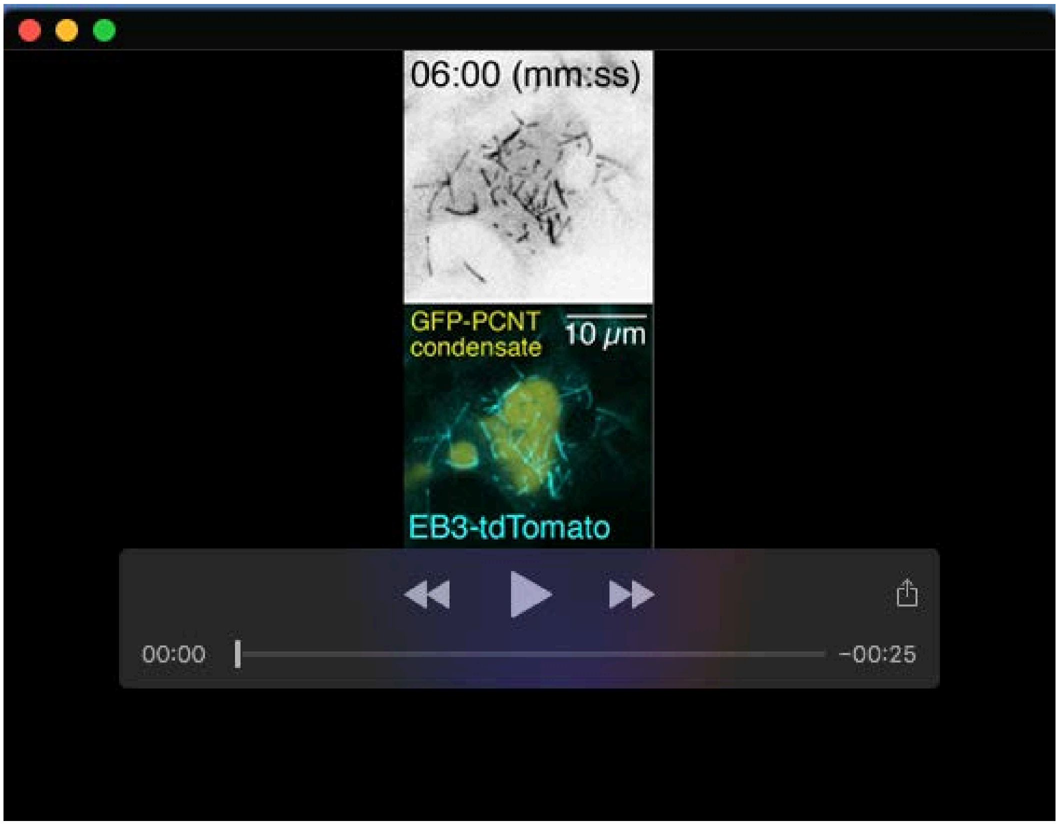

Movie 8. Time-lapse movie of microtubules renucleating from the surface of GFP-PCNT (854-1960) conden-sates in RPE-1 cells. Microtubules, whose plus ends were labeled by EB3-tdTomato (cyan), were emanating from the surface of the PCNT condensate (yellow) after nocodazole was washed out. The whole cell view and individual images of this time series are shown in Fig. 7.

Journal of Cell Science • Supplementary information
